# Modeling the Effect of Environmental Geometries on Grid Cell Representations

**DOI:** 10.3389/fncir.2018.00120

**Published:** 2019-01-14

**Authors:** Samyukta Jayakumar, Rukhmani Narayanamurthy, Reshma Ramesh, Karthik Soman, Vignesh Muralidharan, V. Srinivasa Chakravarthy

**Affiliations:** ^1^Department of Biotechnology, Bhupat and Jyoti Mehta School of Biosciences, Indian Institute of Technology Madras, Chennai, India; ^2^Department of Biotechnology, Rajalakshmi Engineering College, Chennai, India; ^3^Department of Bioengineering, University of California, Berkeley, Berkeley, CA, United States; ^4^Department of Psychology, University of California, San Diego, San Diego, CA, United States

**Keywords:** grid cells, spatial cells, oscillatory path integration, Lateral Anti-Hebbian Network, connected environment, concave environment, convex environment

## Abstract

Grid cells are a special class of spatial cells found in the medial entorhinal cortex (MEC) characterized by their strikingly regular hexagonal firing fields. This spatially periodic firing pattern is originally considered to be independent of the geometric properties of the environment. However, this notion was contested by examining the grid cell periodicity in environments with different polarity (Krupic et al., 2015) and in connected environments (Carpenter et al., 2015). Aforementioned experimental results demonstrated the dependence of grid cell activity on environmental geometry. Analysis of grid cell periodicity on practically infinite variations of environmental geometry imposes a limitation on the experimental study. Hence we analyze the dependence of grid cell periodicity on the environmental geometry purely from a computational point of view. We use a hierarchical oscillatory network model where velocity inputs are presented to a layer of Head Direction cells, outputs of which are projected to a Path Integration layer. The Lateral Anti-Hebbian Network (LAHN) is used to perform feature extraction from the Path Integration neurons thereby producing a spectrum of spatial cell responses. We simulated the model in five types of environmental geometries such as: (1) connected environments, (2) convex shapes, (3) concave shapes, (4) regular polygons with varying number of sides, and (5) transforming environment. Simulation results point to a greater function for grid cells than what was believed hitherto. Grid cells in the model encode not just the local position but also more global information like the shape of the environment. Furthermore, the model is able to capture the invariant attributes of the physical space ingrained in its LAHN layer, thereby revealing its ability to classify an environment using this information. The proposed model is interesting not only because it is able to capture the experimental results but, more importantly, it is able to make many important predictions on the effect of the environmental geometry on the grid cell periodicity and suggesting the possibility of grid cells encoding the invariant properties of an environment.

## Introduction

Spatial navigation is essential for the survival of a mobile organism. Entorhinal cortex (EC), an important cortical area that forms input to the hippocampus, was reported to have neurons known as grid cells which fire when the animal is at points that have a spatially periodic structure (Hafting et al., 2005). Since the periodicity encountered is often hexagonal, these cells are further known as hexagonal grid cells (Hafting et al., 2005). Albeit grid cells were initially discovered in rats (Hafting et al., 2005), these cells have also been reported in mice (Fyhn et al., 2008), bats (Ulanovsky and Moss, 2007; Yartsev et al., 2011), monkeys (Killian et al., 2012), and humans (Jacobs et al., 2013; Moser et al., 2014). Experimental studies in human adults who are at genetic risk for Alzheimer's disease have reported that the neural degeneration originates in the EC, with the loss of grid cell representations causing further impairment of spatial navigation performance of the patient (Kunz et al., 2015).

Preliminary studies on the effects of environmental geometry on spatial cells such as place cells (Barry et al., 2006) and grid cells have been conducted. Place cells are critical for coding the animal's position in space. They fire when the animal is situated in a particular space of the environment known as its firing field (O'Keefe and Dostrovsky, 1971). Remapping of place cells occurred when sufficient changes to the geometry (Lever et al., 2002), color (Bostock et al., 1991), or odor (Anderson and Jeffery, 2003) of the environment were made. Grid cells are equally crucial for spatial navigation by path integration i.e., tracking position by integrating self-motion even without the presence of external sensory landmarks (Hafting et al., 2005; Fuhs and Touretzky, 2006; McNaughton et al., 2006; Burgess et al., 2007; Hasselmo et al., 2007; Fiete et al., 2008; Hasselmo, 2008; Moser et al., 2014; Bush et al., 2015). Grid cells have been proposed to have a role in computing directional vector between the start and goal location (which was termed as vector navigation) that further aids the animal in reaching its goal location (Bush et al., 2015). The variation of the grid scale across the dorsal to ventral medial entorhinal cortex (MEC) axis (Brun et al., 2008; Stensola et al., 2012), acts like a ruler with different resolutions to measure the distance traversed by the animal from its starting location. The features of the grid cells that are stated above help the animal to navigate the environment efficiently.

MEC conveys spatial information from the higher sensory cortical areas to the hippocampus (Barnes et al., 1990; Quirk et al., 1992; Fyhn et al., 2004). It is believed that the dynamic representation of the spatial location of an animal is created and updated by the MEC and the grid cells are a proof of it (Savelli et al., 2008). Hargreaves et al. (2005) initially recorded MEC grid cells from an environment (Hargreaves et al., 2005). Since the size of the environment was relatively small, the neurons did not show obvious grid like firing patterns. It was ambiguous in prior studies whether all spatially modulated cells in the MEC were variants of the grid cells or whether a subset resembled the place cells of the hippocampus (Savelli et al., 2008). Savelli et al. (2008) conducted an experiment where the rats were allowed to forage a small box which was placed inside a larger box. After sometime, the small box was removed from the large box without removing the rats and now the rats foraged the larger box for the rest of the experiment. It was observed that some cells that showed place cell like response in the small box, showed grid cell like response in the larger box and the cells showing boundary cell like response showed no change upon the removal of the small box. From the aforementioned experiments it is possible to draw two inferences: firstly, it suggests that there were two major classes of spatial neurons, the grid cells and the boundary cells. The boundary cells may therefore be binding the grid cell firing to the boundaries of the environment. Secondly, the experiment strongly demonstrated that the spatial firing of the MEC cells was strongly influenced by the boundaries of the environment, in the sense that representation of the MEC cells changed predominantly when the local cues of the environment were altered. In this paper, we are addressing the second inference from a purely computational point of view.

Grid cells were initially considered to be the universal metric for navigation due to their minimal remapping property across the environment (Hafting et al., 2005; Fyhn et al., 2007). But this feature of grid field invariance across the environment was contested by the experiment conducted by O'Keefe (Krupic et al., 2015) wherein rats were allowed to forage inside differently shaped environments such as circle, square, hexagon, and trapezoid. Analysis of grid cell activity in each environment revealed that the hexagonal grid field symmetry was affected by the symmetries of the environmental shape. Circle, the most symmetric environment, had a regular hexagonal firing field. As the number of axes of symmetry dropped, the regular hexagonal firing field started to transform into a skewed hexagonal field. This experimental study pointed out that grid cell firing fields were not invariant with respect to the environment but exhibit a definite dependence on the geometry of the environment.

Another interesting experimental study (Carpenter et al., 2015) considered how grid cells responded when the animal foraged inside similar environments connected by a corridor. A key result of the study was that initially the grid fields in each room had a high spatial correlation between them; as the time progressed, this correlation decreased and the grid fields in the two environments became a continuum, forming a global representation of the connected pair of environments. This study revealed a new face of the grid cell coding, whereby the periodic firing fields of the grid cell could rearrange among themselves to reflect the global shape of connectivity of the environment.

Most of the experimental studies on grid cells were performed on either square or circular environments, and have not explored the rich possibilities of varying environmental geometries. Another study by Stensola et al. (2015) focused on analyzing the shear induced asymmetry on entorhinal grid cells (Stensola et al., 2015). Here, the animal was allowed to explore different square enclosures with a rotational offset which elliptically distorts the grid patterns. This distortion is then analytically reversed by a shearing transformation on the grid patterns explaining the phenomenon of anchoring of grid patterns to specific reference points in the enclosure. Although this study involves exploring the change in grid representation, it does not involve analyzing the representations in different environmental geometries, thereby placing it outside the scope of our current study. Apart from the study of Barry et al. (2007) and Krupic et al. (2015), to the best of our knowledge, no experimental studies have been conducted on grid cells under varying environmental geometries.

Krupic et al. (2014) also studied the changes in grid representations in varying environmental geometries from a computational point of view. The proposed model is not biologically plausible as the firing patterns of grid cells are generated based on the assumption that each of the N-number of fields on an abstract semi-infinite 2-D plane interact with each other via attractive and repulsive forces. These fields are not intrinsically generated by neural dynamics, but are distributed a priori over the space and controlled by the dynamics of the abstract force equation. Theoretical studies have been made on grid cell coding in non-Euclidean space (Urdapilleta et al., 2015) which predicted the transformation of the hexagonal pattern of firing field to heptagonal pattern with the change from Euclidean to non-Euclidean space. But the problem of studying grid cell coding as a function of practically infinite variations of the environmental geometry poses a Himalayan challenge to spatial cell researchers. Hence, we propose to classify environmental geometries into the following five broad categories and study the emergent grid fields using computational modeling.

- Connected environments
- Convex shaped environments
- Concave shaped environments
- Regular polygon environments with varying number of sides
- Transforming the environment

The previously mentioned studies, from a pure computational point of view, would result in a better understanding of the spatial encoding inside the brain generated by the grid cells. We show that our simulations not only explain and confirm earlier studies, but also make a number of testable predictions verifiable by experiments.

## Methods

**Figure 1:**
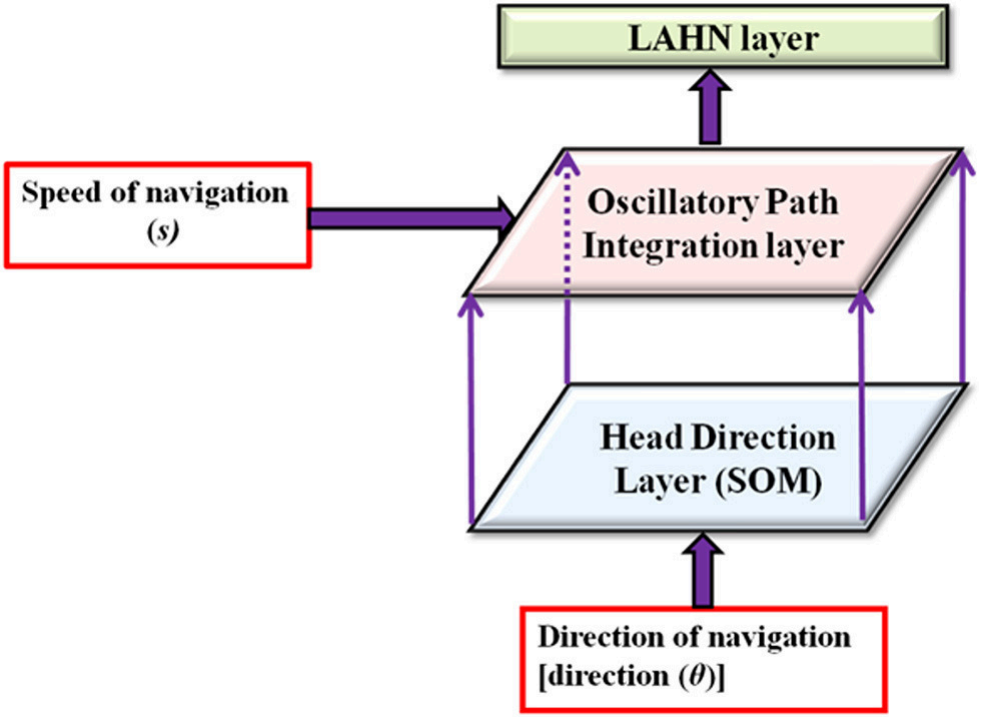
Model architecture. Model receives the velocity information as input and passes through a hierarchy of processing stages such as head direction layer, path integration layer and lateral anti hebbian network (LAHN). Arrows show the direction of flow of information from one hierarchy to the next.

To achieve the goal of studying the effect of environmental geometry on grid cell coding, we used the model as explained below. In this model, the virtual animal is represented as a point in two–dimensional space and is made to forage inside the aforementioned range of environments. Figure 1 shows the model architecture. Values of the parameters used in the equations are given in Table 1.

**Table 1:** Parameter values.

**Parameter**	**Values**
*Ω_*PI*_*	12π rad/s
μ	1
*η_*F*_*	0.01
*η_*L*_*	0.01
β	50
*dt*	0.01 s
*n*	20 (number of neurons in lahn)

In essence, the model has three stages as described below.

### Direction Encoding Stage

The Head Direction (HD) stage is modeled using a two-dimensional layer of neurons whose afferent weight connections are trainable using Hebbian rule. In addition to this, the neuronal layer ensures topography in its representation by training it using Kohonen's Self-Organizing Map (SOM) algorithm (Kohonen, 1982). The network is trained using unit vectors that span the complete 360° angular space. Projection of the animal's current direction on the HD layer forms a neural representation of it and hence forms the directional map (Kohonen, 1982). The response equation of the SOM neuron is given as:

(1) $$\begin{array}{r} \theta_{HD}=\psi^{T}W \end{array}$$

ψ is the two dimensional input given to the SOM such that

ψ = [cos(θ) sin(θ)] where θ is the actual direction of navigation

*W* is the afferent weight matrix of the SOM, where the weight vectors are normalized.

### Oscillatory Path Integration (PI) Stage

This stage consists of a two dimensional array of phase oscillators, which has one-to-one connections with the HD layer. The directional input from Equation (1) is fed to the phase dynamics of the oscillator so that each oscillator corresponding to a specific direction codes for that component of the positional information as the phase of the oscillator. The dynamics of phase oscillator is given as

(2) $$\begin{array}{l} \frac{d(\chi_{u}(i,j))}{dt}=-\chi_{v}(i,j)\left[\Omega_{PI}+\beta s\theta_{HD}(i,j)\right]\\ \;+\chi_{u}(i,j)\left[\mu -(\chi_{u}{\left(i,j\right)}^{2}+\chi_{u}{\left(i,j\right)}^{2})\right] \end{array}$$

(3) $$\begin{array}{l} \frac{d(\chi_{v}(i,j))}{dt}=\chi_{u}(i,j)\left[\Omega_{PI}+\beta s\theta_{HD}(i,j)\right]\\ \;+\chi_{v}(i,j)\left[\mu -(\chi_{u}{\left(i,j\right)}^{2}+\chi_{v}{\left(i,j\right)}^{2})\right] \end{array}$$

χ _u_ and χ_v_ are the *u* and *v* state variables of the PI oscillator.

β *is* the spatial scale parameter.

*s* is the speed of the navigation such that *s* = ||X(t)–X(t−1)|| where X is the position vector of the animal.

μ is the parameter that controls the limit cycle behavior of the oscillator. Here μ is taken as 1.

### Lateral Anti-hebbian Network (LAHN) Stage

LAHN is an unsupervised neural network (Földiák, 1989) that extracts optimal features from the input. The network has 1D array of neurons with lateral inhibitory and afferent excitatory connections. These weight connections are trainable using biologically plausible neural learning rules such as Hebbian (for afferent weights) and Anti-Hebbian (for lateral weights). The lateral inhibitory connections induce competition among the neurons and the afferent Hebbian connections extract principal components from the input (Oja, 1982). This network connectivity hence ensures optimal feature extraction from the input data. It has also been observed that neurons that give rise to grid representations are connected via GABAergic interneurons (Pastoll et al., 2013), thereby establishing inhibitory lateral connections between them as seen in the LAHN layer of the model.

The response of the network is given by the following equation.

(4) $$\begin{array}{r} \xi_{i}\left(t\right)=\overset{m}{\underset{j=1}{\sum }}q_{ij}\chi_{j}\left(t\right)+\overset{n}{\underset{k=1}{\sum }}w_{ik}\xi_{k}\left(t-1\right) \end{array}$$

*q* is the afferent weight connections and *w* is the lateral weight connections.

ξ is the response of the network.

*n* is the total number of neurons in the LAHN layer.

*m* is the dimension of the input.

The afferent connections are updated by a variation of the Hebbian rule and the lateral connections are updated by Anti-Hebbian rule as given below.

(5) $$\begin{array}{r} \Delta q_{ij}=\eta_{F}\left[\chi_{j}\left(t\right)\xi_{i}\left(t\right)-q_{ij}{\xi_{i}}^{2}\left(t\right)\right] \end{array}$$

(6) $$\begin{array}{r} \Delta w_{ik}=-\eta_{L}\xi_{i}\left(t\right)\xi_{k}\left(t-1\right) \end{array}$$

η_*F*_and η_*L*_ are the forward and lateral learning rates, respectively.

It has been proved that training the weights of LAHN using Equations (5) and (6) makes the network weights to converge to the subspace spanned by the principle components (PC) of the input data (Földiák, 1989). We have previously showed that training of LAHN on oscillatory path integration values can potentially give rise to a wide variety of spatial cells (Soman et al., 2018b). Although the LAHN layer in the model exhibits a variety of spatial cells, we primarily focused on the hexagonal grid cells to compare with the experimental results.

### Trajectory Generation

The trajectory is designed using dynamics of curvature constrained motion (Soman et al., 2018b) which is governed by the following equations:

(7) $$\begin{array}{r} \overset{•}{x}\left(t\right)=\sigma \left(t\right)\cos\Theta \left(t\right) \end{array}$$

(8) $$\begin{array}{r} \overset{•}{y}\left(t\right)=\sigma \left(t\right)\sin\Theta \left(t\right) \end{array}$$

(9) $$\begin{array}{r} \sigma \left(t\right)=||X_{pos}-X_{wall}|| \end{array}$$

(10) $$\begin{array}{r} |\overset{•}{\Theta }\left(t\right)|\leq \frac{\gamma \left(t\right)}{\rho } \end{array}$$

*x* and *y* determine the position of the virtual animal in 2d space, while its speed is controlled by σ. To ensure that there is high degree of randomness (Equation 10) when it is far off from the boundary and low randomness when it is close by and to prevent it from crossing the boundary, the speed of the virtual agent is reduced when the virtual animal is close to the border (Equation 9).

The model comprises of a pure Path Integration system (i.e., it integrates velocity information at each point in the trajectory), therefore information given to the system can be treated as a sequence problem where continuous integration of the input takes place. The spatial patterns (output of the system) thus depend highly on the way path integration is performed in the model. In such a system, the pattern of the trajectory matters and also changes when there is a change in the shape of the environment, thereby influencing the activity of the spatial cells. In conclusion, behavioral anisotropy has a profound influence on the spatial representation in the model.

### Quantification of Gridness

The neuronal firing activity is represented in the form of three maps namely, the firing field map of the neuron, firing rate map and the autocorrelation map. Red dots are marked on the positions of the animal's trajectory where the SC layer neuron activity crosses a certain threshold value (ε_*sc*_). The activity (firing rate) of the neuron in its firing field is determined by the firing rate map. In the firing rate map, high activity is indicated by red and no activity by blue.

Hexagonal gridness is quantified by a gridness score value (Hafting et al., 2005) computed from the autocorrelation map which is obtained from the firing rate map using the following equation.

(11) $$r(\tau_{x},\tau_{y})=\frac{M\sum_{x,y}\lambda (x,y)\lambda (x-\tau_{x},y-\tau_{y})-\sum_{x,y}\lambda (x,y)\sum_{x,y}\lambda (x-\tau_{x},y-\tau_{y})}{\sqrt{[M\sum_{x,y}\lambda {\left(x,y\right)}^{2}-[\sum_{x,y}\lambda (x,y){]}^{2}][M\sum_{x,y}\lambda {\left(x-\tau_{x},y-\tau_{y}\right)}^{2}-{\left[\lambda (x-\tau_{x},y-\tau_{y})\right]}^{2}}}$$

*r* is the autocorrelation map.

λ*(x,y)* is the firing rate at (x,y) location of the rate map.

*M* is the total number of pixels in the rate map.

τ_*x*_ and τ_*y*_ corresponds to x and y coordinate spatial lags.

Hexagonal Gridness Score (HGS) is computed as given below.

(12) $$\begin{array}{l} HGS=\min\left[cor(r,r^{60^{0}}),cor(r,r^{120^{0}})\right]\\ \;-\max\left[cor(r,r^{30^{0}}),cor(r,r^{90^{0}}),cor(r,r^{150^{0}})\right] \end{array}$$

### Designing the Environments

#### Generation of Regular Polygons With Varying Number of Sides

The polygons are constructed using a unit circle with center at (0, 0). The circle is then sectored into equal angular separation based on the number of sides given as the input.

The positional coordinates of the points on the unit circle that form the polygon are given by

$$\begin{array}{l} \left[X_{k}\;Y_{k}\right]=\left[\cos(\frac{2\pi k}{n})\sin(\frac{2\pi k}{n})\right] \end{array}$$

Angle separation = 2π/n; *n* = number of sides.

The X_k_ and Y_k_ coordinates are then connected to generate the regular polygon (**Figure 9**).

### Generation of Connected Environments

**Figure 2:**
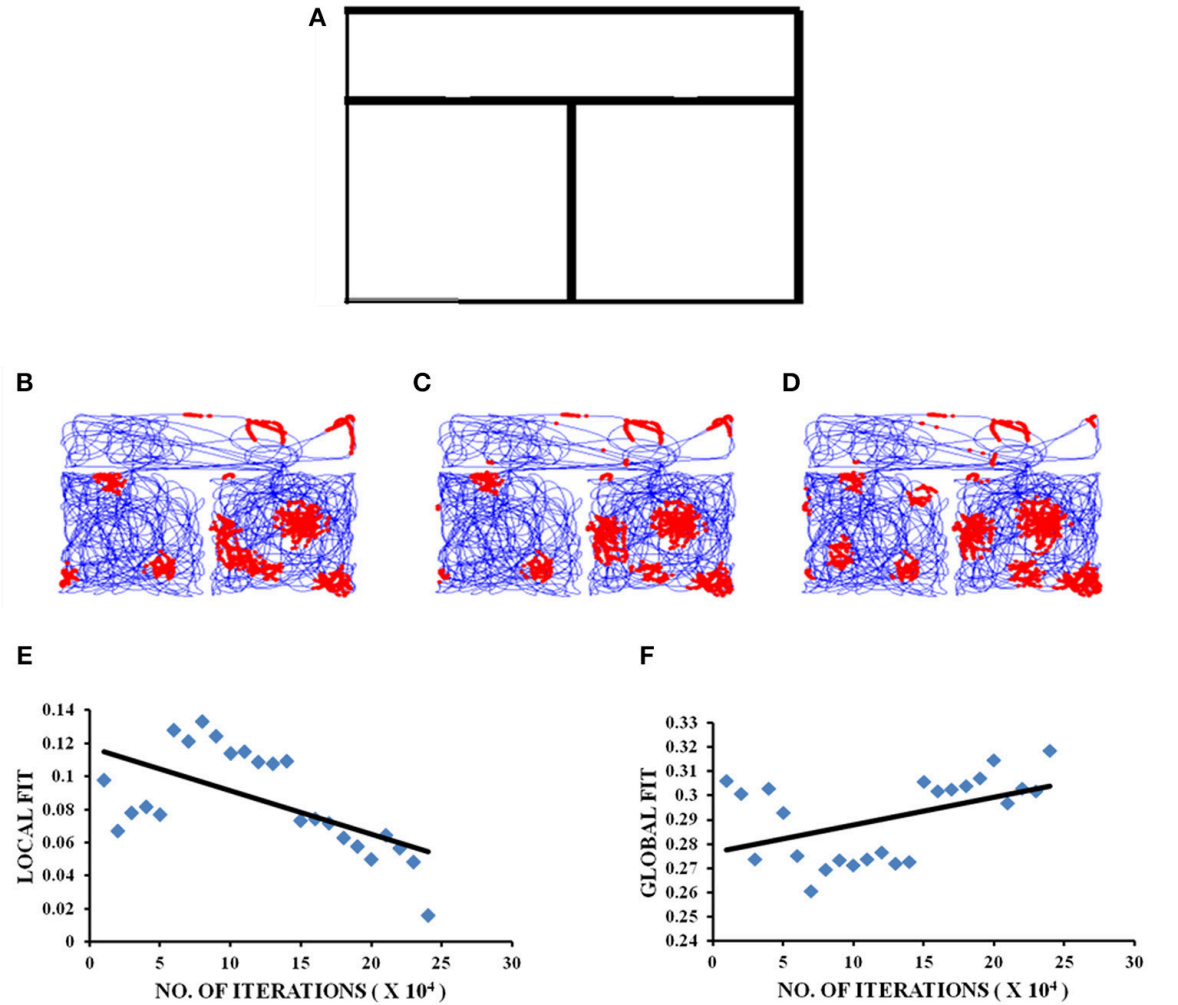
Grid cell firing in square-square connected environment. **(A)** Boundary of square–square connected environment. **(B–D)** represent the firing fields of grid cell neuron for square–square connected environment during different training iterations (beginning, middle, and end, respectively) of the model. **(E,F)** Local and Global HGS values plotted against the no. of training iterations of model.

**Figure 3:**
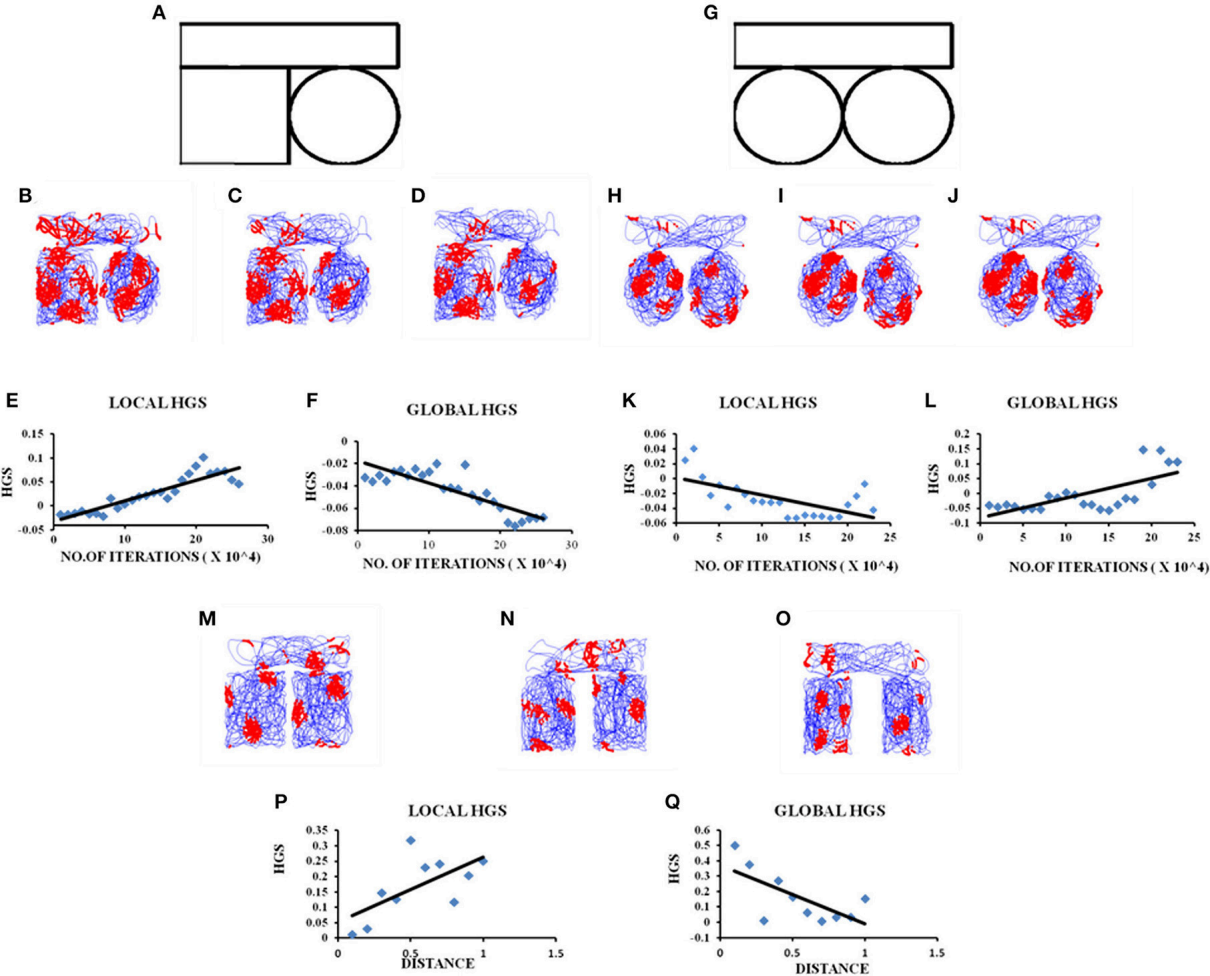
Grid cell firing in circle-circle, square-circle environments and in square–square Connected Environment with increasing distances. **(A)** Boundary of square–circle connected environment. **(B–D)** represent the firing fields of the grid cell in the LAHN for square–circle connected environment at different training iterations (beginning, middle, and end, respectively) of the model. **(E,F)** Local and Global HGS values plotted against the no. of training iterations of model. **(G)** Boundary of circle–circle connected environment. **(H–J)** represent the firing fields of the grid cell in the LAHN for circle–circle connected environment at different training iterations (beginning, middle, and end, respectively) of the model. **(K,L)** Local and Global HGS values plotted against the no. of training iterations of model. **(M–O)** firing field maps (corresponding to end iteration of LAHN training) of the grid cell with respect to distances (d) = 0.1, 0.5, and 1 unit, respectively. **(P,Q)** Local and Global HGS _final_ with respect to the distance between the compartments.

The connected environment used for our study has the same boundary conditions used in the experiment (Carpenter et al., 2015). The two compartments (e.g., square-square) connected via a rectangular corridor are constructed by joining the corner coordinates of the two environments (Figures 2A, 3A,G). For connected environments with varying distances between the two compartments, we introduce a distance parameter “*d*”. The displacement between the two squares is parallel to one side of each of the two square environments (Figures 3M–O).

### Generation of Concave Boundaries

In this category, we consider the annulus, horseshoe and S-shape as instances of concave shapes. To construct an annulus shape, two circles are generated separately (of different radius) and then combined to form two concentric circles (**Figure 5A**). In the case of a horseshoe (**Figure 5B**), two semicircles are concatenated to obtain the shape. The coordinates of both these shapes are determined using the following equations

$$\begin{array}{l} \left[X_{1}\;Y_{1}\right]=\left[r_{1}\text{cos(}\theta \text{)}\;r_{1}\text{sin(}\theta \text{)}\right];\text{corresponds to the outer arc}\\ \left[X_{2}\;Y_{2}\right]=\left[r_{2}\text{cos(}\theta \text{)}\;r_{2}\text{sin(}\theta \text{)}\right];\text{corresponds to the inner arc} \end{array}$$

r_1_ and r_2_ = radii of the outer and inner arcs, respectively, with r_1_ > r_2_. For the annulus, θ varied from 0 to 360° and in the case of horseshoe it varied from 0 to 180°. The S-shaped boundary is generated by concatenating two horseshoe boundaries, with one of the horseshoes inverted to form the S- shape (**Figure 5C**).

### Generation of Transforming Environment

The objective of studying a transforming environment is to see if the network output codes not just the position, but also the global property of the output environment. In the transforming environment, the boundary comprises of a 5 × 2 rectangular boundary (configuration 1) (**Figure 11A**) that evolves over time to a square (configuration 2) of 5 × 5 dimensions (**Figure 11B**). The exploration of the environment by the virtual agent is concurrent with this transformation.

## Results

### Visual Input Is Not Imperative for the Prescribed Model

The model is capable of simulating spatial cell responses even in the absence of any visual cues (Soman et al., 2018b). Furthermore, since it is a velocity driven model, information about the geometry of the environment is implicitly coded in the velocity itself. This is due to the fact that the trajectory of the virtual animal is constrained by the shape of the external environment (Equations (7–10).

### Grid Cell Response to the Shape of Connected Environments

We perform two different studies to understand the grid cell coding that emerges when the animal forages the environments connected by a narrow corridor. In the first study we manipulate the shapes of the connected environments and analyze the grid fields. In the second study we fix the shape but vary the distance between the connected environments.

#### Manipulating the Shapes of the Connected Environments

We simulate connected environments with boundaries and corridor in the same dimensions (the dimension of the square room is 1.8 × 1.8 units and that of the corridor is 0.8 unit)as used in the experimental study (Carpenter et al., 2015). We verify grid cell coding under three schemes such as square–square, square-circle and circle–circle as shown in Figures 2A, 3A,G). The virtual animal is allowed to forage the environment in these three cases. For each case, the model is trained and the resulting grid fields are analyzed as shown in Figures 2, 3.

The local and global fits for the square–square connected environment are calculated in the same manner as mentioned in the experimental paper (Carpenter et al., 2015). As for the other two connected environments (square–circle and circle–circle), the global HGS is computed by calculating the HGS values over the entire connected environment and the local HGS is obtained by calculating the HGS values for the two environments separately and averaging them. In the square–square connected environment, the global fit shows an increasing trend (Figure 2F) with respect to the LAHN training time (Regression analysis: Global fit *R*^2^ = 0.3826, *p* < 0.05). Local fit shows a decreasing trend (Figure 2E; Regression analysis: Local fit *R*^2^ = 0.771, *p* < 0.001).

A similar analysis is performed for connected environments with different shapes such as square–circle. These boundaries are connected in the exact same manner as in the square–square case. In this case, the global HGS shows a reverse trend (Figure 3F) compared to the square–square case (Regression analysis: Global fit *R*^2^ = 0.7042, *p* < 0.001) and the local HGS shows an increasing trend (Figure 3E). (Regression analysis: Local fit *R*^2^ = 0.8152, *p* < 0.001).

We then connect two circles exactly in the same manner as the ones before. Similar analysis is carried out where the global HGS shows an increasing trend (Figure 3L) (Regression analysis: Global fit *R*^2^ = 0.4833, *p* < 0.001) and the local HGS shows a decreasing trend (Figure 3K) (Regression analysis: Local fit *R*^2^ = 0.4071, *p* < 0.001). These trends are similar to the square-square case.

The realignment of the grid fields from a local to global continuum is available for viewing in the Supplementary Material (Videos 1–3).

#### Manipulating the Distance Between the Connected Environments

Experimental studies showed that grid cells were capable of forming coherent global representations in a connected environment (Carpenter et al., 2015). Our next objective is to examine whether this globally representative property of grid firing is retained with increasing distance between the connected environments. To examine this property we connect two square compartments and vary the distance between them (distance (d) ranging from 0.1 to 1 unit, in increments of 0.1). The boundary conditions of the compartments and the corridor are set in the same ratio as in the experiment (Carpenter et al., 2015). The agent is allowed to forage the environment for a period of 10 sessions. Each session consists of five trips and for each session the distance between the two compartments is increased by a value of 0.1.

HGS values (HGS_final_) are computed at the time of convergence of LAHN and the global and local fits are calculated. These scores are taken into account owing to the fact that convergence corresponds to the completion of the training session of the weights. The global HGS values over the distances show a decreasing trend (Figure 3Q; Regression analysis: Global fit *R*^2^ = 0.4585, *p* < 0.05) while the values of the local HGS show an increasing trend (Figure 3P; Regression analysis: Local fit *R*^2^ = 0.4142, *p* < 0.05).

### Grid Cell Response in Convex Shaped Environment

**Figure 4:**
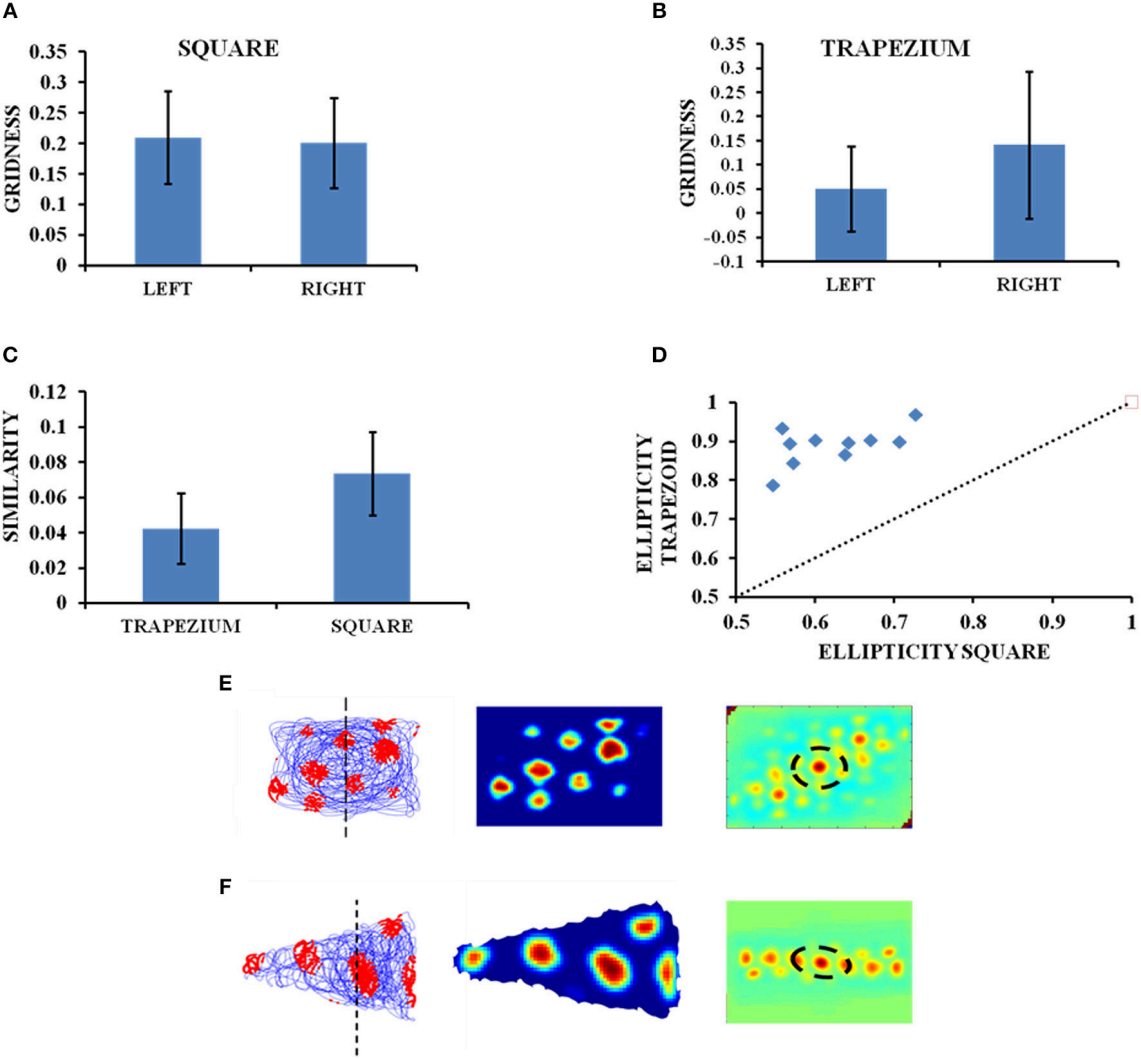
Grid cell firing in square and trapezoid enclosures. **(A,B)** Average HGS values plotted for the left and right orientation of the square and trapezium boundaries. **(C)** Model results—Right and left sides of square are more similar than that of a trapezium. **(D)** Model results—Ellipticity between a square and a trapezoid. **(E,F)** Firing field, firing rate, and autocorrelation maps of the square and trapezoid boundaries, respectively.

The influence of environmental geometry on grid cell symmetry was contested by Krupic et al. (2015), and the notion that grid cells can serve as a universal metric for navigation was challenged. We conduct a similar study using our computational model where we generate square and trapezoidal boundaries in the same dimensions as used in the experiment (Krupic et al., 2015). The HGS values are calculated from the autocorrelation map. Both the square and trapezoid boundaries are divided into two halves of equal areas and analysis is performed to check the similarity in the gridness between the two halves for both the boundaries. The HGS analyses are carried out independently for each side and then for the complete shape in case of both trapezoid and square. The average HGS for the left side of the trapezoid is less compared to its right (left = 0.050763, right = 0.14143; Figure 4B) and is more or less equal for both sides of the square (left = 0.201337, right = 0.20981, Figure 4A). Another study on the similarity between the grid patterns of both the halves in the square and trapezoid enclosures was conducted. It is seen from the Figure 4C that the similarity in grid patterns is high in the square than in the trapezium. Additionally, an analysis to determine the ellipticity of the grid field in the autocorrelogram is conducted. It can be observed that the trapezoid boundary has a higher ellipticity in grid fields than that of a square boundary (Figure 4D).

### Grid Cell Response in Concave Shaped Environment

**Figure 5:**
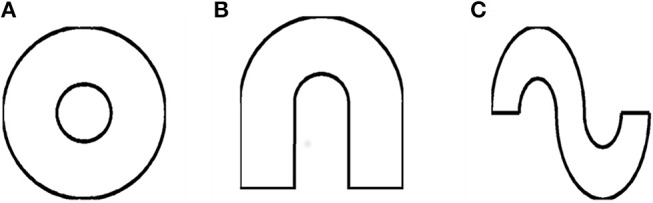
Simulated concave shaped environments **(A)** Annulus, **(B)** Horseshoe, and **(C)** S-Shape, respectively.

A similar study of grid cell spatial coding is conducted using concave shaped environments like horseshoe, annulus and S shape (Figure 5).

#### Horseshoe Shaped Environment

**Figure 6:**
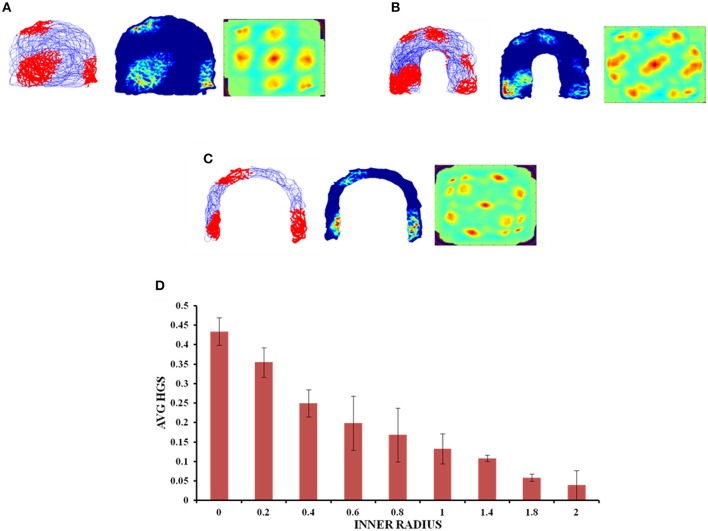
Grid cell responses in horseshoe shaped environment. **(A–C)** Firing field, firing rate, and auto correlation maps of horseshoe boundary with varying inner radii of 0, 0.8, and 2, respectively. **(D)** Average HGS values vs. inner radii of horseshoe shaped environment.

The inner radius (*r*) of the horseshoe is varied from 0 to 2 units with a step size of 0.2. The horseshoe boundary with *r* = 0 approximates a semicircle. The virtual agent is made to traverse the environment. Firing activity of the grid cells under various *r* values is shown in Figures 6A–C. HGS values show a decreasing trend (Figure 6D) as the inner radius of the horseshoe is increased (Single factor ANOVA, *p* < 0.001).

#### Annulus Shaped Environment

**Figure 7:**
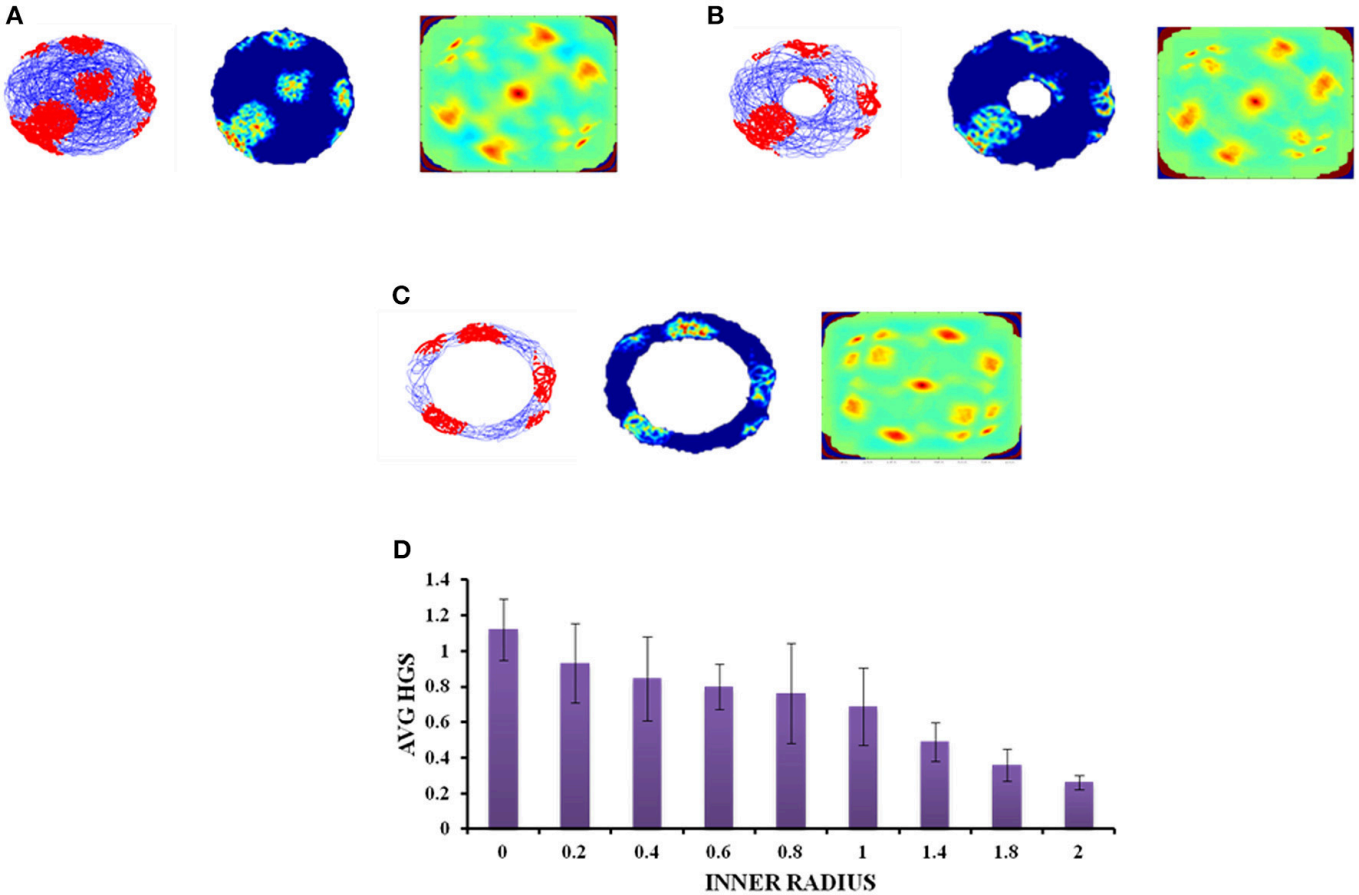
Grid cell response in annulus shaped environment. **(A–C)** Firing field, firing rate, and auto correlation maps of grid activity in the annulus environment with varying inner radii of 0, 0.8, and 2, respectively. **(D)** Average HGS values vs. varying inner radii of annulus.

A similar analysis is performed with the second type of concave boundary i.e., annulus shaped environment. Annulus with inner radius 0 approximates to a circular boundary. Firing activity of the grid cells under various *r* values is shown in Figures 7A–C. The HGS values are found to have the same decreasing trend as that of horseshoe, as the inner radii of the annulus is increased (Single factor ANOVA, *p* < 0.001) (Figure 7D).

#### S Shaped Environment

**Figure 8:**
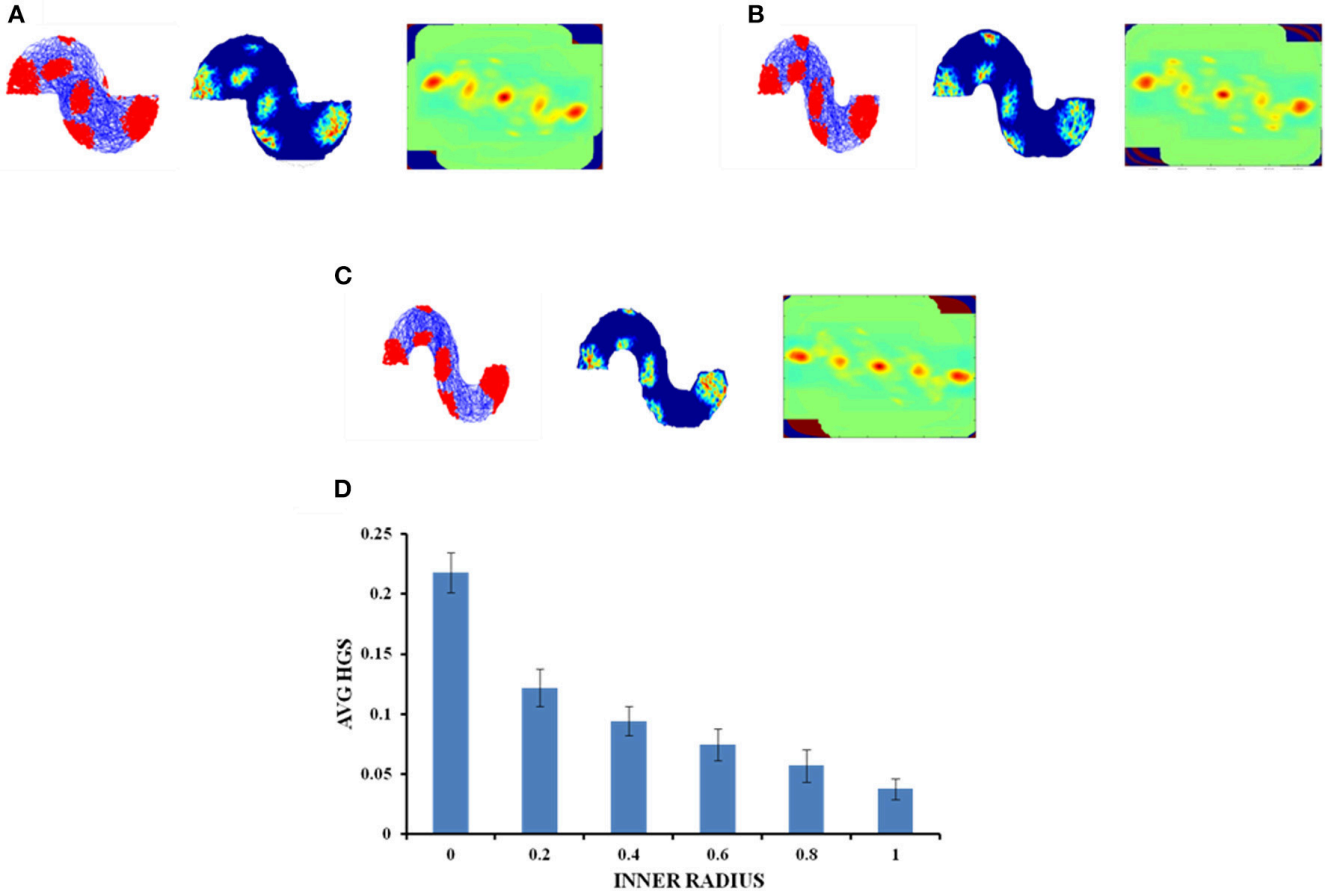
Grid cell response in S-Shape Boundary. **(A–C)** Firing field, firing rate, and auto correlation maps of S shape boundary with varying inner radii of 0, 0.8, and 2, respectively. **(D)** Average HGS values vs. varying inner radii of S- Shape.

In case of an environment like the S shape, where two similar horseshoes are concatenated at a common end, it is found that as the inner radius of the S shape increased from 0 to 1 unit with a step size of 0.2, the HGS values show a decreasing trend (Single factor ANOVA, *p* < 0.001) (Figure 8D).

### Grid Cell Response to Increasing Lines of Symmetry in the Environment

**Figure 9:**
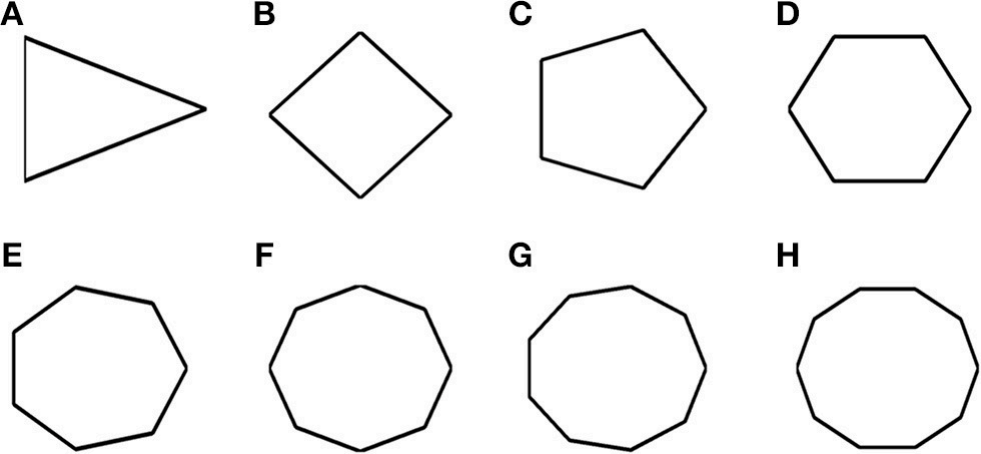
Boundaries of n- sided polygons. **(A–H)** Environmental shapes used for the analysis of grid cell coding with respect to the number of sides of the polygon; arranged in the order of triangle, square, pentagon, hexagon, heptagon, octagon, nonagon, and decagon (from left to right).

Most of the grid cell experimental recordings were carried out either in square or circular shaped environments (Krupic et al., 2015). Here we address the problem of grid cell coding for environments in the shape of n-sided regular polygons. Specifically we consider the range of n from 3 to 10. This study would give an understanding on how the grid cell code will vary if the number of sides increases and the polygon approximates a circle (a regular polygon with infinite sides). In other words, it is analogous to the study of the influence of environmental symmetry on the grid cell code. Figure 9 shows the simulated environments used for this study.

**Figure 10:**
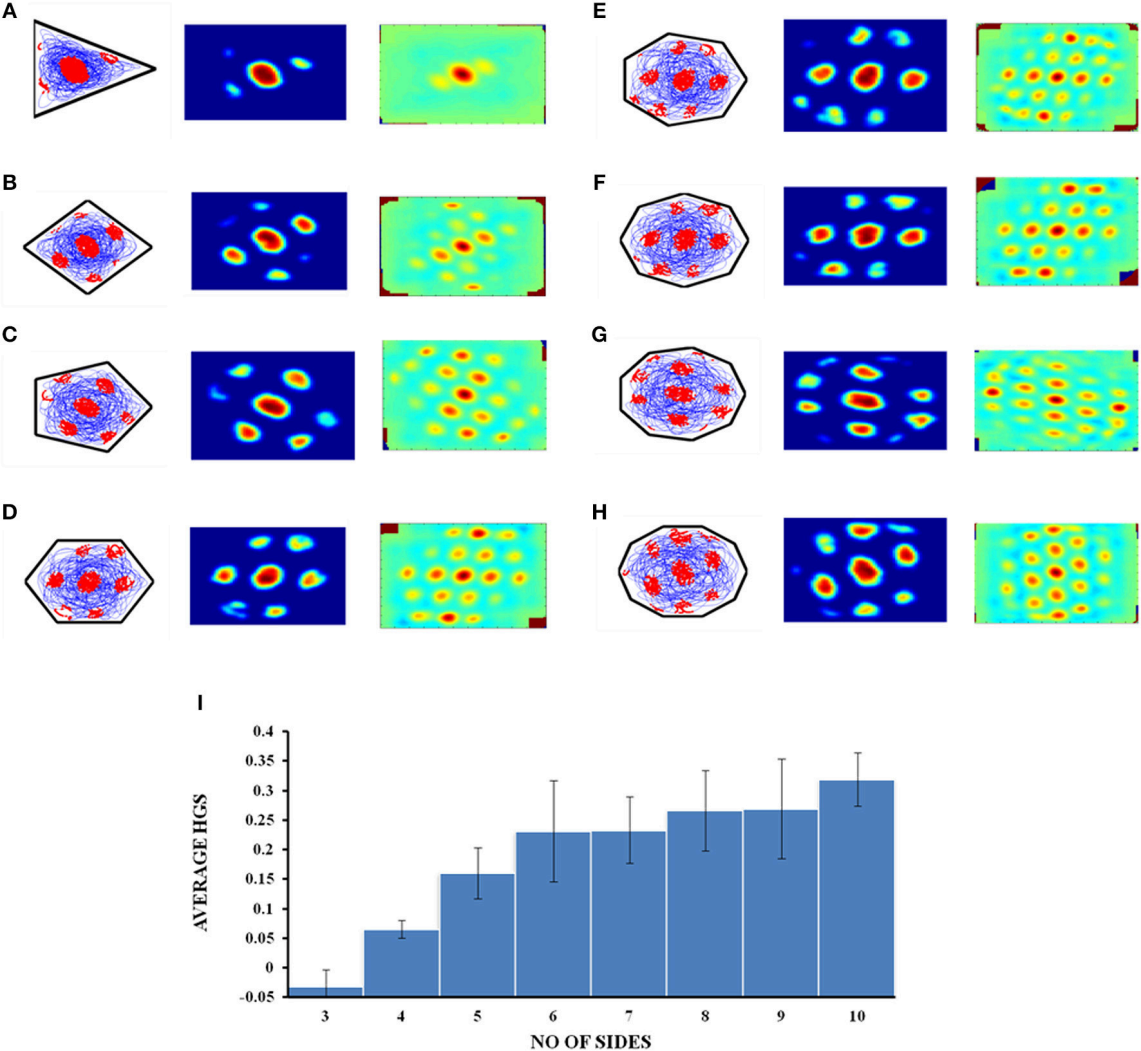
Grid cell firing in regular polygons. **(A–H)** Firing field, firing rate map and autocorrelogram (from left to right) of triangle, square, pentagon, hexagon, heptagon, octagon, nonagon, and decagon shapes, respectively. **(I)** The average HGS value of the grid field obtained for each polygon vs. the number of sides of the polygon. The plot shows an increasing trend as the polarity of the environment decreases.

The virtual animal is then allowed to forage inside these shapes and the resultant trajectory is given as input to the model. Figures 10A–H show the resultant firing field, firing rate and autocorrelation maps of the respective polygons. We compute the HGS values from the spatial autocorrelograms. It is observed that the HGS values shows an increasing trend with respect to the number of sides of the polygon (Single factor ANOVA, *p* < 0.001) (Figure 10I).

### Deciphering the Global Feature of the Environment From LAHN Activity

The results described so far point to the fact that the simulated grid neuron is sensitive to a global feature of an environment such as its shape. To make a more general statement, we need to show that the LAHN neurons code for a more invariant and global feature of an environment, in addition to just the local features such as position or displacement. To numerically prove this qualitative statement, the virtual animal is allowed to forage in a rectangular configuration. Over the course of time, where a rat was made to forage inside multi the environment is transformed into a square. To prove the above hypothesis, we show that the LAHN neural responses have information to classify the environment—Rectangle vs. Square. We implement this classifier using a Multi-Layer Perceptron (MLP).

The MLP is used here to classify the configuration (either rectangle or square) based on the activity of the LAHN. The MLP is trained using the standard back propagation algorithm (LeCun, 1988) with a single hidden layer of neurons [see Supplementary Material for MLP training (B)]. The classification accuracy after training comes to 74.74%. To confirm for the global invariant feature in the LAHN, we trained another MLP with partially learned LAHN and its classification accuracy comes to 52.33%. Furthermore, it can be observed from **Figure 12C** that the Mean Square Error of the network output with respect to the untrained LAHN input is 0.5307 (configuration 1) and 0.2854 (configuration 2); while for a trained LAHN input it is 0.1503 (configuration 1) and 0.1359 (configuration 2). This proves that as the LAHN learns the representations of the animal's navigating space, it encodes both local (like displacement, position) and global (like the environmental configuration) spatial features. For efficient navigation, both of these features are pertinent and the animal must be performing Simultaneous Localization And Mapping (SLAM) (Milford and Wyeth, 2010).

## Discussion

Grid cell firing fields, characterized by their hexagonal spatial periodicities, are considered to serve as a universal metric for spatial navigation. This notion was contested by some experimental studies (Krupic et al., 2015) showing the dependence of grid cell coding on the environmental shape. However, the experimental studies have limitations with regard to the grid cell recording under different environmental shapes. This forms the motivation of the present paper which seeks to study, using computational modeling, grid cell activity under different environmental geometries without any limitations that plague experimental efforts. Since we chose to study the grid cell activity under various environmental conditions, we systematically divided the simulations into five categories such as connected environments, convex shaped environments, concave shaped environments, regular polygonal environments (with varying number of sides), and transforming environment. Finally, through the numerical analysis and MLP classifier we show the potentiality of the spatial cells to encode the global and invariant feature of the environment rather than local features like position, displacement etc.

The experimental study conducted by Carpenter et al. (2015) where the rat foraged between two similar square boxes connected via a corridor, forms the special case of the formerly stated modeling study where the shape is square-square and distance is zero. The modeling results concur with the experimental results (Figure 2). In the experimental case it was observed that initially the grid cell firing was controlled by the local cues, in the sense that the firing replicated between the two compartments. However, with further exploration, the similarities between the grid firing fields of the compartments decreased, suggesting that with increasing trials, global cues controlled the firing of grid cells. This trend is captured in the model (Figure 2). Hence, the aforementioned simulation and further analysis of the grid cell activity in the connected environments form a viable empirical study.

In the connected environment study, we manipulated the shapes of the connected regions from a square-square to a square-circle and circle-circle boundary. HGS values are computed while the model is undergoing training. The interesting result is that in the case of identical connected environments, such as square-square and circle-circle, the global HGS values (HGS value computed from the connected environment as a whole) show an increasing trend and the local HGS values (average of the HGS values computed from each connected region separately) show a decreasing trend with respect to the training time of the model. This means that as the animal gets more and more familiar with the environment (with increasing training sessions in the model), the grid fields start to realign themselves and form a continuum in the case of similar shaped connected environments. In order to determine whether the global representation of the connected environments depend on the similarity between the two connected boundaries, we performed a similar analysis in a square-circle environment. On the contrary, in this case it showed a negative trend in the global HGS and a positive trend in the local HGS value. Thus, it is possible to infer from the grid cell HGS variation whether the animal is exploring in a similar or dissimilar connected environment. This is a novel insight into the grid cell code purely from the computational point of view, and an easily testable prediction for future experiments.

After studying the grid cell coding scheme with respect to the shapes of the connected environments, we delved into the dependence of grid cell coding scheme on the distance between the connected environments. This study, along with the formerly stated one, is pertinent especially with regard to large scale navigation where the animal is not restricted to just one environment but shuttles between multiple environments of different shapes at different locations. Hence to get an insight on the grid cell coding with respect to the distance between the connected environments, we simulated a connected environment (square-square) and varied the distance between the two compartments of the environment. Our hypothesis was that since grid cells code for the distance traveled by the animal (O'Keefe and Burgess, 2005) (due to its regularly periodic hexagonal firing field), the distance between the connected environments should also be reflected in its activity. On performing the analysis, the variation in the global HGS values show a decreasing trend and local HGS values show an increasing trend with increasing distance between environments. This variation in the gridness score points out the possibility that grid cells encode for the global distance between the environments and this information is pertinent to large scale navigation. It is observed from the grid cell representations that the two compartments, even though connected, are treated as independent at larger distances.

Hence at the outset, when the distance between the compartments is minimal, the representation is more global as opposed to local. As the distance between the two compartments increases, the grid cells seem to lose their ability to form global representations and the firing becomes localized to their respective compartments. From the above simulations, the inference is that grid cells may not code just for distance but also for the entire structure of the environment. The methods defined previously can be easily extended to the case of connected environments with more than two components. We can consider a network of environments with complex spatial arrangements and connectivity. It would be interesting to study the evolution of local vs. global organization of the grid fields in such systems. In addition to the spatial arrangements of the environments in such complex systems, the frequency of visitation of that agent to individual components may also determine the overall grid field organization. Such studies might pave way to the formulation of deep laws that govern the spatial encoding of brain in compound environments with complex navigational patterns followed by the agent.

Oscillatory path integration stage of the model is vital to capture the results that are indicated above. Here, the position is encoded as the phase of the oscillator (Equations 2, 3). If a grid cell is activated at one point in space in one case (for instance, in connected environments separated by distance d_1_) and not activated at the same point in the second case (distance d_2_), the reason must be that the afferent input to the grid cell from the oscillators is different (Equation 4) in both the cases. Different configurations of the environment make the oscillator code for the same position at different phases of the oscillator. Also, since position is encoded as a periodic quantity at this oscillatory stage [as it does in oscillatory interference model (Burgess et al., 2007)], this periodicity is reflected in the spatial firing fields of the grid cell in the LAHN.

The objective of the next simulation study is to essentially capture the results of the experimental work by Krupic et al. (2015), where a rat was made to forage inside multiple boundaries of different shapes such as circle, square, trapezium and hexagon. This study explained the permanent effect exerted by the environmental geometry on grid cell firing and grid field symmetry. To determine the impact of environmental characteristics on homogeneity and symmetry of grid patterns, the grid firing in two shapes such as square and trapezium was analyzed. It was found that in a highly polar environment like a trapezium there was a decrease in the regularity of the hexagon (reflected in the HGS score) and the pattern becomes highly elliptical across the entire enclosure. To estimate the regularity of grid patterns, the trapezoid and square were divided into two parts of equal area and the firing fields on both the sides were compared. The autocorrelation maps showed that there was a strong difference in local spatial structures between the two sides of a trapezoid unlike a square wherein they were highly similar. The gridness of left (narrower) side of the trapezoid was found to be low when compared to its right (broader) side. Also when the square and trapezoid boundaries were compared as a whole, the latter had a lower gridness. This was because when a trapezoid is divided into two, the left side resembled a triangle and the right side, a square.

The simulation results are consistent with the experimental data (Figure 4). We performed the comparative study as mentioned above using our model i.e., between both sides of the trapezoid and square and between both the shapes as whole and obtained congruent results (Figure 4). From the firing rate map (Figures 4E,F) and autocorrelation map (Figures 4E,F) we are able to see that the left side of the trapezoid has less local spatial structure compared to its right side. In the case of a square, little difference is observed between its two halves. A study on the similarity of the grid patterns between the two halves of both the trapezoid and square enclosures, respectively, revealed that the patterns are more similar in the square enclosure than in the trapezoid enclosure. In addition to this the ellipticity of the grid fields from the autocorrelation maps of the trapezoid and square boundaries were obtained and plotted as observed in the Figure 4D. It can be inferred that the grid fields in the trapezoid boundary are more elliptical than the grid fields in the square boundary and this as well is in congruence with the experimental results (Krupic et al., 2015).

Since the model successfully captured the experimental results (Krupic et al., 2015) in the convex shaped environments such as square and trapezoid, we extended our study by varying the number of sides of a regular polygon. The aim of this study is to understand the effect of the number of lines of symmetry in the environment on the grid fields. We found that the HGS values show an increasing trend with respect to the number of sides of the regular polygon (Figure 10I). In other words, higher symmetry in the environment leads to higher HGS values. Hence we predict that the HGS score should be maximum for a circular environment (where the number of lines of symmetry is infinite). It was also observed from the experiment (Krupic et al., 2015), that the circular boundary is considered to be highly unpolarized when compared to all the other boundaries and hence showed high gridness scores.

The above experimental study focuses on explaining the influence of the polarity of the environment on grid representations. But this study takes into account only discreet environments such as square, hexagon, circle and trapezium. The N-Sided Polygon study that we have conducted is a generalization of this experimental study where we dynamically analyse the polarity induced asymmetry in grid representations by gradually increasing the number of sides of the environment (maintaining a continuum) until the outline approximates a circle (considered to be highly non-polar).

Since the real world navigation occurs in environments with arbitrary shapes, we conducted the simulation on concave shapes too. We considered concave shapes such as horseshoe, annulus and S-shaped environments (Figure 5). As the inner radius of the horseshoe increased, the hexagonal grid representation in the auto correlation map appeared to lose its regularity. This is captured by the decreasing HGS values as shown in the graph (Figure 6D). The same trend is observed for the other two concave boundaries as well, i.e., annulus and S-Shape (Figures 7D, 8D). It can be observed that, since the annulus with inner radius 0 approximates a circular boundary, its HGS value tends to be the highest. It can be noted that as the inner radii of the aforementioned concave environments increase, the space available for the virtual animal to traverse shrinks. This reduced availability of space may be the reason behind the deviation from the hexagonal spatial coding of the grid cell, as reflected in the reduced HGS values across all the three concave shapes. This can be experimentally tested in many ways by implementing environments similar to the ones that we have simulated in this study (Figures 6–8) with less space given to the animal for exploration. There is also a need to develop a strong mathematical framework that explains the relationship between the observed spatial coding and the geometry of the environment. Hence the proposed modeling study gives a new dimension to the grid cell coding with a good number of testable predictions.

**Figure 11:**
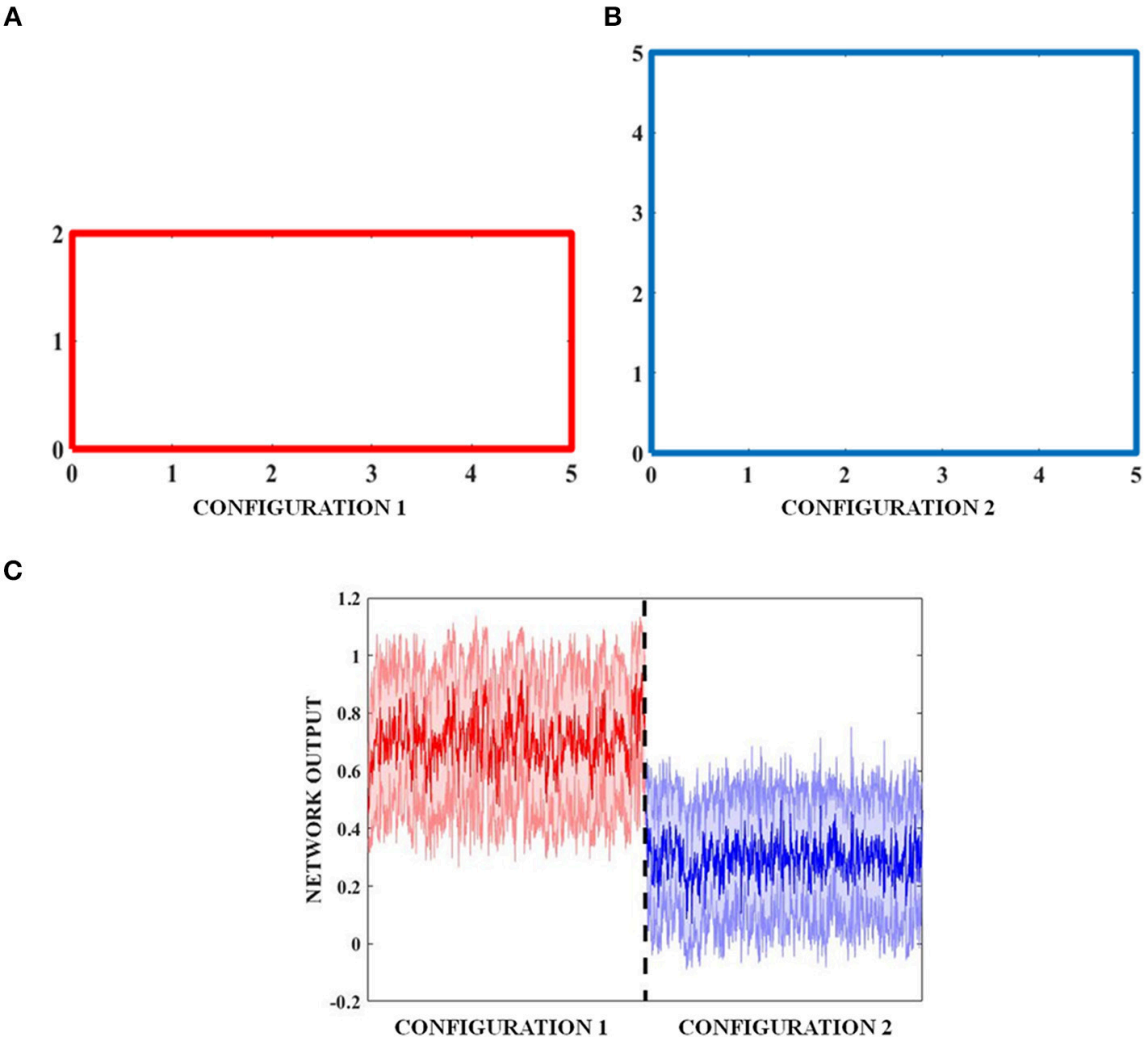
Transforming environment. **(A)** Rectangle **(B)** Temporal transformation of the rectangle to a square by increasing its breadth, and **(C)** shaded plot of the classification of the two boundaries by MLP.

**Figure 12:**
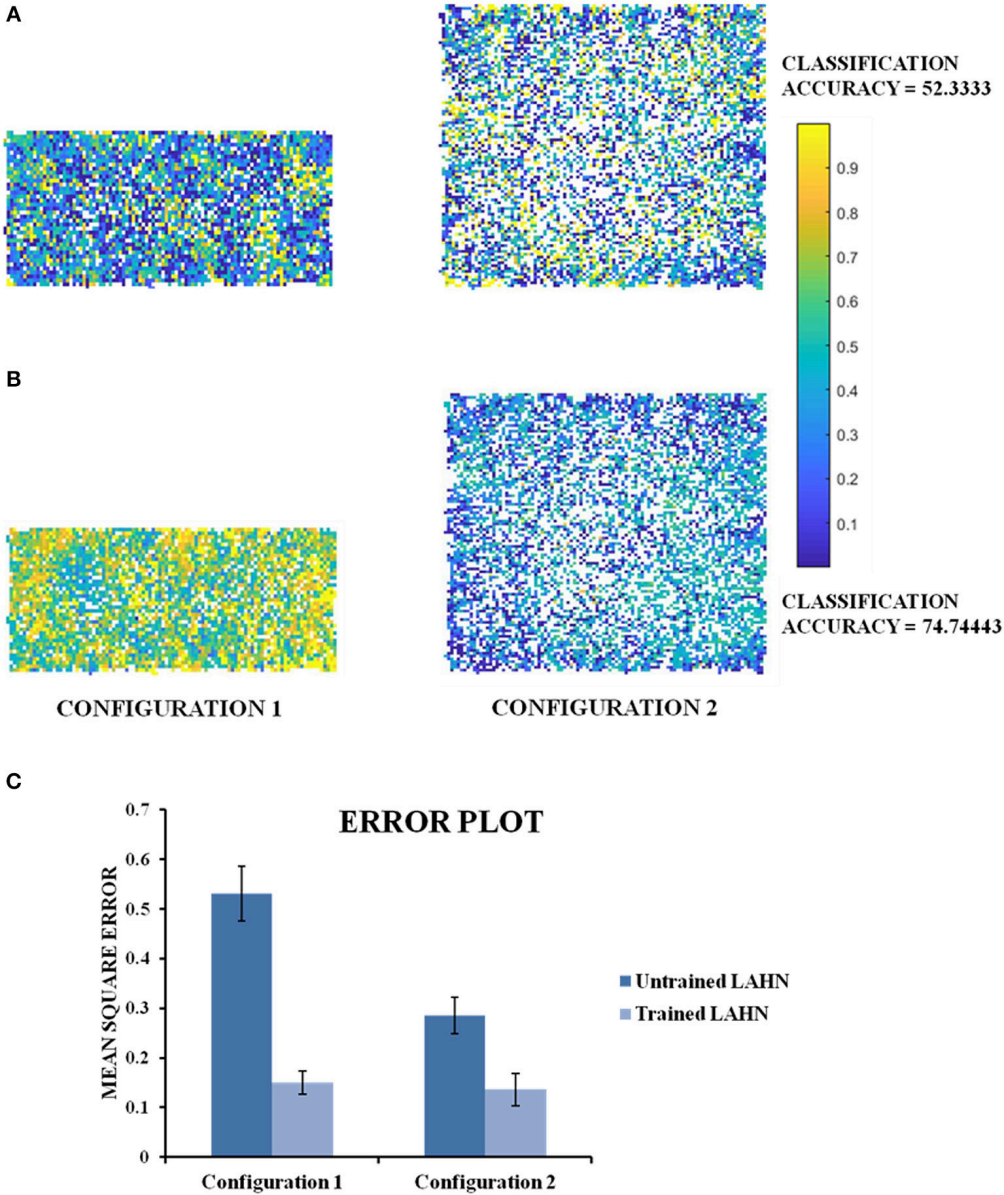
Classification maps of the MLP output. **(A)** MLP classification maps using initial training periods of LAHN as input. It gives a classification accuracy of 52.33% **(B)** MLP classification maps using fully trained LAHN as input. It gives a classification accuracy of 74.74%.Yellow and dark blue denote configuration 1 and configuration 2, respectively. The intermediate color gradient represents output of the network activity between 0 and 1. **(C)** Mean Square Error of the MLP network output.

In all the studies stated above, we looked for grid fields only for the sake of comparison with empirical evidence. However, we hypothesize that it is not just the grid cells but the entire LAHN that implicitly codes for the global structure of the environment rather than just local structure of the space in which the animal navigates. To underpin this, we decoded the LAHN neural information using MLP whose precision was measured in terms of classification accuracy. We constructed a transforming environment (rectangle to square) and collected the spatial cell responses which were fed as input to the MLP network to check if the LAHN was able to account for this transformation. At any given time, for a given position (x, y) of the virtual animal, the MLP is able to decipher the information about the configuration it had traversed in Figure 11C. This is denoted by the classification accuracy obtained from MLP. A high classification accuracy of 74.74% (Figure 12B) is observed on using trained LAHN input. Conversely when partially trained LAHN is used, the classification accuracy decreased to 52.33% (Figure 12A). This serves as a proof-of-principle that global information is encoded in the population activity of LAHN.

## Model Predictions

In our connected environment study, the model was able to show that the locally and globally representing properties of the grid cells are sensitive to the shape of the environments that are connected with each other. We believe that this could be verified by connecting environments of different shapes in a similar fashion as in Carpenter et al.'s experiment (Carpenter et al., 2015) and analyzing how the representations emerge as the animal forages in this context. This would in turn elucidate the underpinnings of the global pattern of the grid cell that allows it to be a spatial metric (McNaughton et al., 2006; Fiete et al., 2008; Buzsáki and Moser, 2013). Moreover, testing the global and local representing properties of grid cells in connected environments by varying the distance between the compartments, will offer a holistic idea of the factors that these properties of grid cells are sensitive to.

We hypothesize that the LAHN layer in our model approximates a population of cells present in the Hippocampus and the medial Entorhinal Cortex thus focussing on population activity instead of single cell activity. This ensemble of neurons in the LAHN thus holds global information regarding the environmental geometry. The above prediction can be proved empirically by conducting experiments in which the rat is allowed to forage in a square boundary, one side of which is gradually extended until it approximates a rectangle without any interruption to the animal's navigation during the transformation (similar to the transforming environment study). As the rat is navigating in this environment, the collective activity of a population of cells from the hippocampus and medial entorhinal cortex can then be recorded instead of single cell recording. These neural signals can then be decoded using algorithms such as Bayesian decoders (Kloosterman, 2011; Kloosterman et al., 2013) to decipher the shape of the environment rather than just the position information.

## Future Directions

In the model, we give direct velocity inputs to the path integration layer which is further fed to the LAHN layer where spatial cells emerge. A more biologically plausible approach would be to account for the representation of motion-related inputs driven by the locomotion of the animal instead of providing explicit position coordinates or velocity inputs. The motion-related information is conferred to the nervous system by the sensory streams that include vision and proprioception. Although the model accounts for spatial cell responses even in the absence of visual cues, the presence of visual input has been proved to offer more stability to the responses of the spatial cells (Soman et al., 2018a). In the light of this view, it would be more interesting to study the extent of stability that the sensory inputs (particularly vision) offer to the spatial cells when the global characteristics of the environment changes. Furthermore, we would also like to decode the complete boundary information of complex environments (contours, mazes etc.,) using the collective response of the LAHN layer in the model.

## Data Accessibitlity

The simulation code is available at the ModelDB database (http://senselab.med.yale.edu/ModelDB/showModel.cshtml?model=240118) Access code: Env_Grid_Model.

## Author Contributions

All authors contributed equally to the work. RN and SJ performed coding, trajectory generation, analysis of the model, and manuscript preparation. RR performed trajectory generation and analysis of the model. KS and VM performed designing the model, coding, analysis of the model, and manuscript preparation. VC performed designing the model and manuscript preparation.

### Conflict of Interest Statement

The authors declare that the research was conducted in the absence of any commercial or financial relationships that could be construed as a potential conflict of interest.

## Abbreviations

EC: Entorhinal Cortex
HD: Head Direction
HGS: Hexagonal Gridness Score
LAHN: Lateral Anti-hebbian Network
MEC: Medial Entorhinal Cortex
PCA: Principal Component Analysis
PI: Path Integration
SOM: Self Organizing Map
MLP: Multi-Layer Perceptron.

## References

[B1] Anderson M. I. Jeffery K. J. (2003). Heterogeneous modulation of place cell firing by changes in context. J. Neurosci. 23, 8827–8835. 10.1523/JNEUROSCI.23-26-08827.2003 14523083 PMC6740394

[B2] Barnes C. A. McNaughton B. L. Mizumori S. J. Leonard B. W. Lin L. H. (1990). Chapter Comparison of spatial and temporal characteristics of neuronal activity in sequential stages of hippocampal processing. Prog. Brain Res. 83, 287–300. 10.1016/S0079-6123(08)61257-1 2392566

[B3] Barry C. Hayman R. Burgess N. Jeffery K. J. (2007). Experience-dependent rescaling of entorhinal grids. Nat. Neurosci. 10, 682–684. 10.1038/nn1905 17486102

[B4] Barry C. Lever C. Hayman R. Hartley T. Burton S. O'Keefe J.. (2006). The boundary vector cell model of place cell firing and spatial memory. Rev. Neurosci. 17, 71–97. 10.1515/REVNEURO.2006.17.1-2.71 16703944 PMC2677716

[B5] Bostock E. Muller R. U. Kubie J. L. (1991). Experience-dependent modifications of hippocampal place cell firing. Hippocampus 1, 193–205. 10.1002/hipo.450010207 1669293

[B6] Brun V. H. Solstad T. Kjelstrup K. B. Fyhn M. Witter M. P. Moser E. I.. (2008). Progressive increase in grid scale from dorsal to ventral medial entorhinal cortex. Hippocampus 18, 1200–1212. 10.1002/hipo.20504 19021257

[B7] Burgess N. Barry C. O'Keefe J. (2007). An oscillatory interference model of grid cell firing. Hippocampus 17, 801–812. 10.1002/hipo.20327 17598147 PMC2678278

[B8] Bush D. Barry C. Manson D. Burgess N. (2015). Using grid cells for navigation. Neuron 87, 507–520. 10.1016/j.neuron.2015.07.006 26247860 PMC4534384

[B9] Buzsáki G. Moser E. I. (2013). Memory, navigation and theta rhythm in the hippocampal-entorhinal system. Nat. Neurosci. 16, 130–138. 10.1038/nn.3304 23354386 PMC4079500

[B10] Carpenter F. Manson D. Jeffery K. Burgess N. Barry C. (2015). Grid cells form a global representation of connected environments. Curr. Biol. 25, 1176–1182. 10.1016/j.cub.2015.02.037 25913404 PMC4425461

[B11] Fiete I. R. Burak Y. Brookings T. (2008). What grid cells convey about rat location. J. Neurosci. 28, 6858–6871. 10.1523/JNEUROSCI.5684-07.2008 18596161 PMC6670990

[B12] Földiák P. (1989). Adaptive network for optimal linear feature extraction, in Proceedings of the IEEE/INNS International Joint Conference on Neural Networks, Vol. 1 (Washington, DC; New York, NY: IEEE Press), 401–405.

[B13] Fuhs M. C. Touretzky D. S. (2006). A spin glass model of path integration in rat medial entorhinal cortex. J. Neurosci. 26, 4266–4276. 10.1523/JNEUROSCI.4353-05.2006 16624947 PMC6674007

[B14] Fyhn M. Hafting T. Treves A. Moser M. B. Moser E. I. (2007). Hippocampal remapping and grid realignment in entorhinal cortex. Nature 446, 190–194. 10.1038/nature05601 17322902

[B15] Fyhn M. Hafting T. Witter M. P. Moser E. I. Moser M. B. (2008). Grid cells in mice. Hippocampus 18, 1230–1238. 10.1002/hipo.20472 18683845

[B16] Fyhn M. Molden S. Witter M. P. Moser E. I. Moser M. B. (2004). Spatial representation in the entorhinal cortex. Science 305, 1258–1264. 10.1126/science.1099901 15333832

[B17] Hafting T. Fyhn M. Molden S. Moser M. B. Moser E. I. (2005). Microstructure of a spatial map in the entorhinal cortex. Nature 436, 801–806. 10.1038/nature03721 15965463

[B18] Hargreaves E. L. Rao G. Lee I. Knierim J. J. (2005). Major dissociation between medial and lateral entorhinal input to dorsal hippocampus. Science 308, 1792–1794. 10.1126/science.1110449 15961670

[B19] Hasselmo M. E. (2008). Grid cell mechanisms and function: contributions of entorhinal persistent spiking and phase resetting. Hippocampus 18, 1213–1229. 10.1002/hipo.20512 19021258 PMC2614862

[B20] Hasselmo M. E. Giocomo L. M. Zilli E. A. (2007). Grid cell firing may arise from interference of theta frequency membrane potential oscillations in single neurons. Hippocampus 17, 1252–1271. 10.1002/hipo.20374 17924530 PMC2408670

[B21] Jacobs J. Weidemann C. T. Miller J. F. Solway A. Burke J. F. Wei X. X.. (2013). Direct recordings of grid-like neuronal activity in human spatial navigation. Nat. Neurosci. 16, 1188–1190. 10.1038/nn.3466 23912946 PMC3767317

[B22] Killian N. J. Jutras M. J. Buffalo E. A. (2012). A map of visual space in the primate entorhinal cortex. Nature 491, 761–764. 10.1038/nature11587 23103863 PMC3565234

[B23] Kloosterman F. (2011). Analysis of hippocampal memory replay using neural population decoding. Neuronal Network Anal. 67, 259–282. 10.1007/7657_2011_8

[B24] Kloosterman F. Layton S. P. Chen Z. Wilson M. A. (2013). Bayesian decoding using unsorted spikes in the rat hippocampus. J. Neurophysiol. 111, 217–227. 10.1152/jn.01046.2012 24089403 PMC3921373

[B25] Kohonen T. (1982). Self-organized formation of topologically correct feature maps. Biol. Cybernet. 43, 59–69. 10.1007/BF00337288

[B26] Krupic J. Bauza M. Burton S. Barry C. O'Keefe J. (2015). Grid cell symmetry is shaped by environmental geometry. Nature 518, 232–235. 10.1038/nature14153 25673417 PMC4576734

[B27] Krupic J. Bauza M. Burton S. Lever C. O'Keefe J. (2014). How environment geometry affects grid cell symmetry and what we can learn from it. Phil. Trans. R. Soc. B 369:20130188. 10.1098/rstb.2013.0188 24366142 PMC3866452

[B28] Kunz L. Schröder T. N. Lee H. Montag C. Lachmann B. Sariyska R.. (2015). Reduced grid-cell-like representations in adults at genetic risk for Alzheimer's disease. Science 350, 430–433. 10.1126/science.aac8128 26494756

[B29] LeCun Y. (1988). A theoretical framework for back-propagation, in Proceedings of the 1988 Connectionist Models Summer School, CMU, eds Touretzky D. Hinton G. Sejnowski T. (Pittsburgh, PA: Morgan Kaufmann), 21–28.

[B30] Lever C. Wills T. Cacucci F. Burgess N. O'Keefe J. (2002). Long-term plasticity in hippocampal place-cell representation of environmental geometry. Nature 416, 90–94. 10.1038/416090a 11882899

[B31] McNaughton B. L. Battaglia F. P. Jensen O. Moser E. I. Moser M. B. (2006). Path integration and the neural basis of the 'cognitive map'. Nat. Rev. Neurosci. 7, 663–678. 10.1038/nrn1932 16858394

[B32] Milford M. Wyeth G. (2010). Persistent navigation and mapping using a biologically inspired SLAM system. Int. J. Robot. Res. 29, 1131–1153. 10.1177/0278364909340592

[B33] Moser E. I. Roudi Y. Witter M. P. Kentros C. Bonhoeffer T. Moser M. B. (2014). Grid cells and cortical representation. Nat. Rev. Neurosci. 15, 466–481. 10.1038/nrn3766 24917300

[B34] Oja E. (1982). Simplified neuron model as a principal component analyzer. J. Math. Biol. 15, 267–273. 10.1007/BF00275687 7153672

[B35] O'Keefe J. Burgess N. (2005). Dual phase and rate coding in hippocampal place cells: theoretical significance and relationship to entorhinal grid cells. Hippocampus 15, 853–866. 10.1002/hipo.20115 16145693 PMC2677681

[B36] O'Keefe J. Dostrovsky J. (1971). The hippocampus as a spatial map. Preliminary evidence from unit activity in the freely-moving rat. Brain Res. 34, 171–175. 10.1016/0006-8993(71)90358-1 5124915

[B37] Pastoll H. Solanka L. van Rossum M. C. Nolan M. F. (2013). Feedback inhibition enables theta-nested gamma oscillations and grid firing fields. Neuron 77, 141–154. 10.1016/j.neuron.2012.11.032 23312522

[B38] Quirk G. J. Muller R. U. Kubie J. L. Ranck J. B. (1992). The positional firing properties of medial entorhinal neurons: description and comparison with hippocampal place cells. J. Neurosci. 12, 1945–1963. 10.1523/JNEUROSCI.12-05-01945.1992 1578279 PMC6575876

[B39] Savelli F. Yoganarasimha D. Knierim J. J. (2008). Influence of boundary removal on the spatial representations of the medial entorhinal cortex. Hippocampus 18, 1270–1282. 10.1002/hipo.20511 19021262 PMC3007674

[B40] Soman K. Muralidharan V. Chakravarthy V. S. (2018a). A model of multisensory integration and its influence on hippocampal spatial cell responses. IEEE Transac. Cogn. Dev. Syst. 10, 637–646. 10.1109/TCDS.2017.2752369

[B41] Soman K. Muralidharan V. Chakravarthy V. S. (2018b). A unified hierarchical oscillatory network model of head direction cells, spatially periodic cells and place cells. Eur. J. Neurosci. 47, 1266–1281. 10.1111/ejn.13918 29575125

[B42] Stensola H. Stensola T. Solstad T. Frøland K. Moser M. B. Moser E. I. (2012). The entorhinal grid map is discretized. Nature 492, 72–78. 10.1038/nature11649 23222610

[B43] Stensola T. Stensola H. Moser M. B. Moser E. I. (2015). Shearing-induced asymmetry in entorhinal grid cells. Nature 518, 207–212. 10.1038/nature14151 25673414

[B44] Ulanovsky N. Moss C. F. (2007). Hippocampal cellular and network activity in freely moving echolocating bats. Nat. Neurosci. 10, 224–233. 10.1038/nn1829 17220886

[B45] Urdapilleta E. Troiani F. Stella F. Treves A. (2015). Can rodents conceive hyperbolic spaces? J. Roy. Soc. Interface 12:20141214. 10.1098/rsif.2014.1214 25948611 PMC4590491

[B46] Yartsev M. M. Witter M. P. Ulanovsky N. (2011). Grid cells without theta oscillations in the entorhinal cortex of bats. Nature 479, 103–107. 10.1038/nature10583 22051680

