# Supplementary material for: Modeling the Effect of Environmental Geometries on Grid Cell Representations
Source: Front Neural Circuits. 2019 Jan 14;12:120. doi: 10.3389/fncir.2018.00120 (PMC6339930; doi:10.3389/fncir.2018.00120)
Supplement: Supplementary file 4 [file Data_Sheet_1.docx]

Supplementary Material

**Modeling the Effect of Environmental Geometries on Grid Cell Representations**

Samyukta Jayakumar, Rukhmani Narayanamurthy, Reshma Ramesh, Karthik Soman, Vignesh Muralidharan, and V. Srinivasa Chakravarthy^*^

*** Correspondence:** V. Srinivasa Chakravarthy: [schakra@iitm.ac.in](mailto:schakra@iitm.ac.in)

1. **Supplementary Movie**

Gradual transformation of grid cell firing with prolonged training sessions in the model, Related to Results.

Video1. The movie shows the evolution of the firing field map of the grid cells in a Square – Square Environment.

Video2. The movie shows the evolution of the firing field map of the grid cells in a Square – Circle Environment.

Video3. The movie shows the evolution of the firing field map of the grid cells in a Circle – Circle Environment.

1. **Training the MLP**

Multi-Layer Perceptron (MLP) is an artificial neural network that is used to classify non-linear data. It is trained using supervised learning and is carried out through backpropagation algorithm. We use MLP to investigate whether the LAHN response could capture the invariant information about the configuration of the environment. Here, the MLP serves as a decoder with a converging architecture comprising of one hidden layer with 25 neurons. The input and output layers contain 40 neurons and 1 neuron respectively.

The LAHN is trained over the trajectory of the virtual animal in the transforming environment and its responses were collected. We then generated a 6 Hz theta oscillation, whose peaks are used to threshold the spatial cell response collected from the neurons in the LAHN layer. This is then given as the input to the MLP.

1. **Implementing noise in the model**

In order to check the consistency of the model in the presence of a noisy signal input, we simulated the model in the presence of Gaussian noise that is added to the path integration layer. A similar analysis of determining the HGS (Hexagonal Grid Score) of the grid cells in a square shaped boundary is conducted. Two inferences are made from the obtained results:

1. The model is able to capture similar results even in the presence of a noisy signal input.
2. The HGS decreases with increase in the noise factor added to the path integration layer as shown in the graph below.

**Figure 1.** The bar graph shows the effect of a noisy signal input on the HGS of the grid cells in a square shaped boundary. It can be observed that as the Gaussian noise input to the path integration layer increases, the HGS decreases and the firing fields of the grid cells get distorted when the noisy input is increased beyond the SD of 1x.

1. **Grid cells with different phase and orientation**

The model is a conglomeration of ‘*n*’ neurons that is therefore capable of producing ‘*m*’ number of grid cells each having a different phase and orientation with respect to others. The figure below shows 5 different types of grid cells with varying phase and orientations from the same LAHN layer.

**Figure 2.** 5 different grid cells obtained from the model with different phase and orientation.

1. **Speed of the virtual agent**

Training in the model was performed with a dt = 0.01 seconds for ~250,000 epochs, thus with a total training time of 2500 seconds or 41 minutes. Speed is a variable given to the model and a histogram of the speed data incorporated is shown in the figure below.


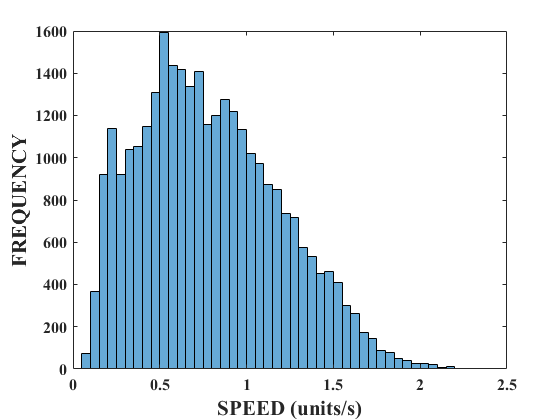


**Figure 3.** Histogram of the speed of the virtual agent in the model. It is evident that speed is a variable with a high frequency between ~0.5 and 0.6 units/s.

1. **Periodic Border – like cells**

Since the trajectory generation depends on the boundary of an environment, some information with respect to the border of the environment is captured by the neurons in the model, thus giving rise to certain periodic border-like cells (cells that fire along the boundary of the environment) as shown in the figure below. We would like to mention here that these simulated neurons are not similar to the empirically reported border cells. The simulated neurons have periodic firing fields near the vicinity of the border and also it does not have any preferential border in the environment. Due to these contrasting features, we name it as periodic border like neurons.

**Figure 4.** Periodic Border-like cells obtained from the model. (A,B,C) – are the firing field, firing rate and autocorrelation maps respectively.

1. **Time Occupancy**

Following is the time occupancy figure that shows the number of times the virtual agent has crossed a particular position in the environment.


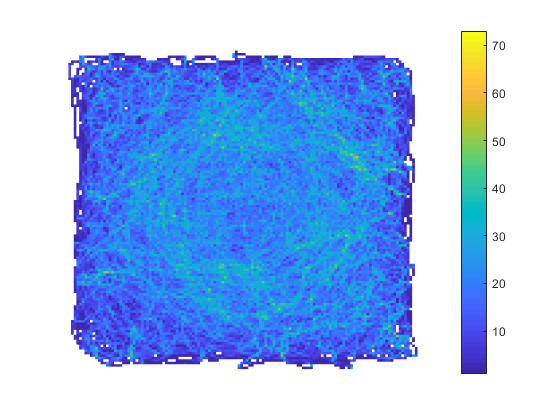


**Figure 5.** Time occupancy plot. The dark blue represents the coalesced trajectories over 10 runs and the cyan represents the number of times the virtual agent has crossed a particular position in the square boundary. The white patches show the unvisited places of the animal.

1. **Local and Global fit for the square-circle and circle-circle connected environments**

To provide validation for the results that were obtained in terms of hexagonal gridness score that showed an increasing trend for local grid representation and decreasing trend for global grid representation in the square-circle case and the vice versa of decreasing local and increasing global HGS for the circle-circle case, we calculated the global and local fit scores for the two environments, in a manner identical to the experiment by Carpenter et al ([Carpenter, Manson et al. 2015](#_ENREF_1))


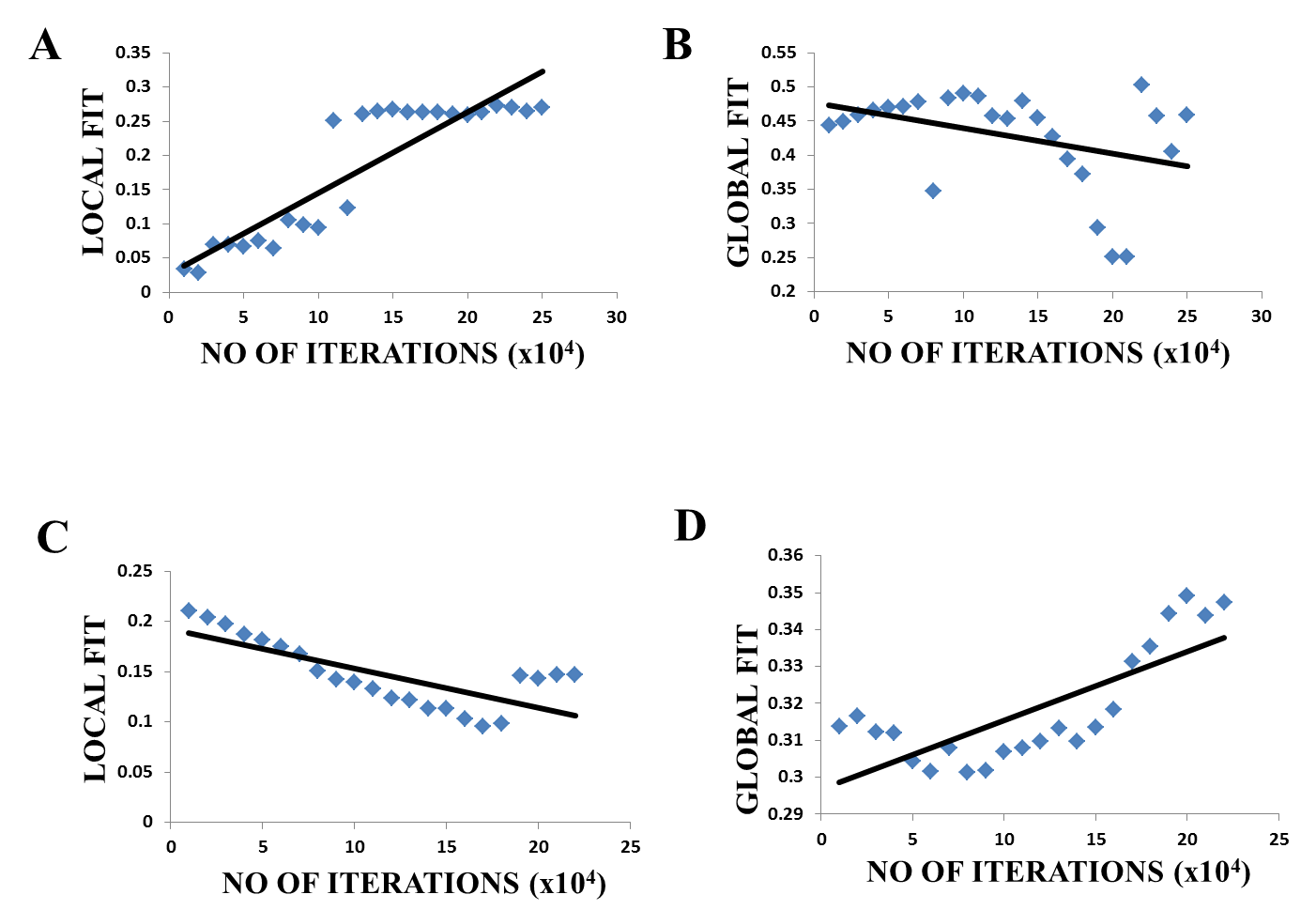


**Figure 6.** Local fit and Global fit. (A, B) – represent the local and global fit against the number of LAHN training iterations in a Square-Circle connected environment. (C, D) – represent the local and global fit against the number of LAHN training iterations in a Circle-Circle connected environment.

1. **Grid score analysis in the polygons of equal area**

All the polygons simulated in this study have an area approximately close to 3 except triangle, square and pentagon. Hence we simulated a triangular and pentagonal enclosure with area of 3 units and analyzed the grid scores. As expected, we observed that as the area increased, the number of grid fields spanning the environments also increased thereby leading to an increase in the average grid scores. Although the grid scores increased, the trend observed as in our original study was conserved, i.e. the average grid scores increased as the number of sides increases. Thus, the grid representations formed are independent of the area and dependent of the boundary restrictions imposed by the environment.


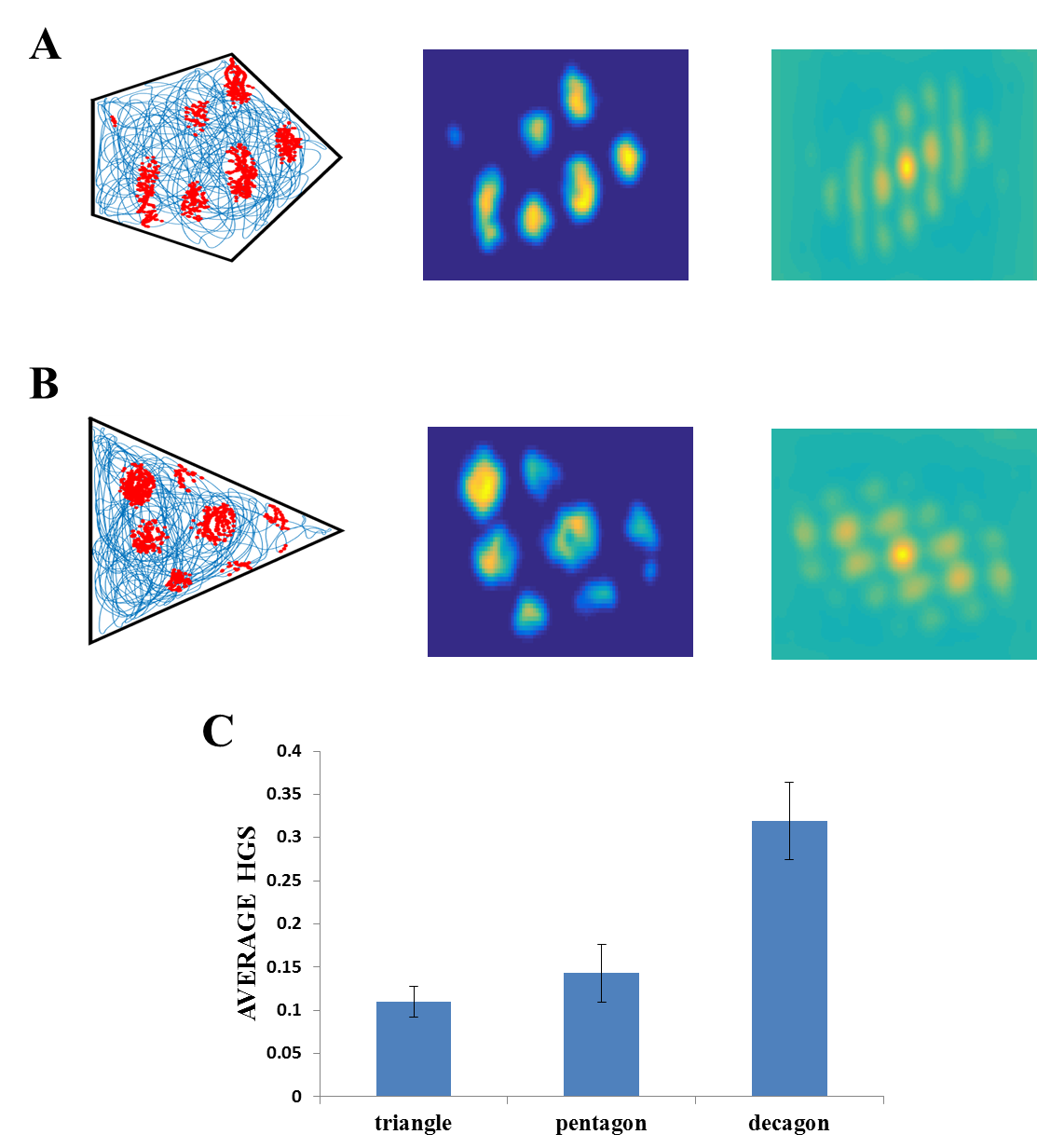


**Figure 7.** Polygons. (A, B) – Firing field, firing rate map and autocorrelogram (from left to right) of triangle and pentagon respectively. (C) the average HGS value of the grid field obtained for each polygon versus the number of sides of the polygon. The plot shows an increasing trend as the environment becomes less polarized.

**References**

Carpenter, F., D. Manson, et al. (2015). "Grid cells form a global representation of connected environments." Current Biology **25**(9): 1176-1182.
